# P2X7 Mediates ATP-Driven Invasiveness in Prostate Cancer Cells

**DOI:** 10.1371/journal.pone.0114371

**Published:** 2014-12-08

**Authors:** Ying Qiu, Wei-hua Li, Hong-quan Zhang, Yan Liu, Xin-Xia Tian, Wei-Gang Fang

**Affiliations:** 1 Key Laboratory of Carcinogenesis and Translational Research, Ministry of Education, Peking University Health Science Center, Beijing, 100191, China; 2 Department of Pathology, School of Basic Medical Sciences, Peking University Health Science Center, Beijing, 100191, China; 3 Department of Anatomy, Histology and Embryology, Peking University Health Science Center, Beijing, 100191, China; University Paris Sud, France

## Abstract

The ATP-gated P2X7 has been shown to play an important role in invasiveness and metastasis of some tumors. However, the possible links and underlying mechanisms between P2X7 and prostate cancer have not been elucidated. Here, we demonstrated that P2X7 was highly expressed in some prostate cancer cells. Down-regulation of P2X7 by siRNA significantly attenuated ATP- or BzATP-driven migration and invasion of prostate cancer cells in vitro, and inhibited tumor invasiveness and metastases in nude mice. In addition, silencing of P2X7 remarkably attenuated ATP- or BzATP- driven expression changes of EMT/invasion-related genes Snail, E-cadherin, Claudin-1, IL-8 and MMP-3, and weakened the phosphorylation of PI3K/AKT and ERK1/2 in vitro. Similar effects were observed in nude mice. These data indicate that P2X7 stimulates cell invasion and metastasis in prostate cancer cells via some EMT/invasion-related genes, as well as PI3K/AKT and ERK1/2 signaling pathways. P2X7 could be a promising therapeutic target for prostate cancer.

## Introduction

Prostate cancer is one of the most common malignant tumors, and also one of the leading causes of male cancer related death worldwide [1]. Most of the patients do not die of local primary tumor, but of distant metastases. The process of cancer metastasis consists of sequential and interrelated events [2]. On one hand, cancer cells can acquire migratory and invasive capabilities by epithelial mesenchymal transition (EMT), which enables them to acquire the capacity to infiltrate surrounding tissues and to ultimately metastasize to distant sites [3]. On the other hand, interactions between cancer cells and tumor microenvironment are essential for the metastatic dissemination of tumors cells [4]. Our previous studies have focused on an important tumor microenvironment molecule, adenosine 5′-triphosphate (ATP).

In addition to having an intracellular role in cell metabolism as an energy source, ATP has been widely accepted as an extracellular signaling molecule, and can be released to extracellular environment in both physiological and pathological situations such as neurotransmission, tissues damage, et al [5], [6], [7]. It is now clear that ATP is one of the abundant biochemical components of the tumor microenvironment and plays a key role in host-tumor interaction. Extracellular ATP acts as a signaling molecule through interaction with P2 receptors and mediates a variety of biological processes such as cell proliferation, migration and invasion [8], [9], [10]. P2 receptors are divided into two distinct families: G protein coupled P2Y receptors (P2Y1, 2, 4, 6, 11, 12, 13 and 14) and ligand-gated cation permeable channel P2X receptors (P2X1-7) [11]. A number of P2 receptors have been reported to be expressed in prostate cancer cells [10], [12]. Our previous study demonstrated that extracellular ATP was an important pro-invasive agent and P2Y2 was one of the key receptors which mediated ATP-promoted migration and invasion of prostate cancer cells [10]. However, we also found that ATP-mediated pro-invasiveness cannot be completely abolished after maximal silencing of P2Y2, indicating that some other receptor subtype(s) might be involved in this process. Among the P2X receptor family, P2X7 is the latest cloned member [13], and was initially considered to be a cytolytic receptor since its prolonged activation leads to cell death [14]. Recently, it was found that P2X7 was abundantly expressed in cancer cells of leukemia, neuroblastoma, melanoma, as well as in prostate, breast and thyroid cancer [15], [16], [17], [18], [19], and was even proposed to be a biomarker for early stage cancer [17], [20], [21]. Furthermore, activation of P2X7 was reported to have anti-apoptotic effects, stimulate tumor cell growth [22], [23], and even to promote cell invasiveness in some cancer cells [24], [25], which is contradictory to the initial assumption that P2X7 was a “death receptor”. In this study, we aimed to investigate the role of P2X7 in the invasion and metastasis of prostate cancer, and to reveal the underlying mechanisms.

## Materials and Methods

### Chemicals and antibodies

ATP, BzATP, KN62, LY294002, U0126 and Digitonin were obtained from Sigma (St Louis, MO, USA). Rabbit anti-P2X7 antibody (#APR-008) was obtained from Alomone Labs (Jerusalem, Israel), and antibodies of β-actin (#TA-09), ERK1/2(#SC-94) and E-cadherin (#SC-7870) were obtained from Santa Cruz Biotechnology (Santa Cruz, CA, USA). Antibodies of Snail (#3895S), Claudin-1 (#4933P), p-AKT (#4056S), AKT (#4691S), and p-ERK1/2 (#9101S) were purchased from Cell Signaling Technology (Danvers, MA, USA). HRP-conjugated goat anti-mouse (#ZB-2305) and goat anti-rabbit IgG (#ZB-2301) were obtained from OriGene (Maryland, USA). Fluo-4AM (#F14201) was obtained from Molecular Probes (Eugene, OR, USA). HBSS (#14025092) was purchased from Invitrogen (Carlsbad, CA, USA).

### Cell lines and culture conditions

The 1E8 and 2B4 cells were derived from the PC-3M human prostate carcinoma cell line. 1E8 was highly metastatic, whereas 2B4 was non-metastatic [26]. 22RV1 prostate cancer cell line and BPH1 were purchased from American Type Culture Collection. All cells were cultured in RPMI 1640 (GIBCO, Grand Island, NY, USA) supplemented with 10% fetal bovine serum in a humidified atmosphere containing 5% CO_2_ at 37°C.

### Reverse transcription and real-time PCR

Total RNA was extracted using Trizol reagent (Invitrogen) following manufacturer’s instructions. 2 µg of total RNA was reversely transcribed into cDNA using MMLV reverse transcriptase (Promega, Madison, Wisconsin, USA), and real-time PCR was performed using ABI StepOne Real-Time PCR System (Applied Biosystems, USA). The specific primers were purchased from Invitrogen and listed in S1 Table. The thermal cycle conditions were as follows: 10 min at 95°C, 30 cycles of 15 s at 95°C and 1 min at 60°C. Relative gene expression levels, normalized to β-actin expression, were calculated using the 2^−△△Ct^ method [27].

### Cell lysis and western blot analysis

Whole cell lysate was extracted with RIPA buffer (Applygen Technologies Inc, Beijing, China) containing protease inhibitors and phosphatase inhibitors (Roche, Mannheim, Germany). The concentration of protein was determined using a BCA reagent (Applygen Technologies Inc, Beijing, China). Equal amounts of protein were separated by SDS-PAGE gel and transferred onto nitrocellulose membranes (Bio-Rad, Hercules, CA, USA), which were incubated at 4°C overnight with primary antibodies against P2X7 (1∶200), E-cadherin (1∶500), Claudin-1 (1∶1000), Snail (1∶1000), β-actin (1∶1000), ERK1/2 (1∶1000), AKT (1∶1000), p-ERK1/2 (1∶1000) or p-AKT (1∶1000) respectively. Immunoreactive proteins were visualized by chemiluminescence (Applygen Technologies Inc) and quantified by densitometry analysis using Quantity One software (Bio-Rad, Hercules, CA, USA).

### Enzyme-linked immunosorbent assay (ELISA)

Cells were cultured in the presence or absence of ATP, and the supernatant was collected after different treatment. The supernatant was stored at −80°C after centrifuging at 12000 rpm for 15 min at 4°C. The IL-8 and MMP-3 protein levels were measured separately using the IL-8 ELISA kit (Invitrogen, Carlsbad, CA, USA) and MMP-3 ELISA kit (Boster, Wuhan, China) following the manufacturer’s instructions. The concentration of IL-8 and MMP-3 were determined by comparing absorbance with the standard protein, and then normalized to total protein.

### Cell transfection

For transient gene silencing, two distinct siRNA oligonucleotides targeting P2X7 were used with sequences as follows: P2X7 siRNA1, 5′-CTAGGATAATGTCCAACTAAA-3′ and P2X7 siRNA2, 5′-CACAGTGTCTTTGACACCGCA-3′. A scramble siRNA was used as negative control. 1E8 and 2B4 prostate cancer cells were transfected with the above siRNA using Lipofectamine RNAi Max (Invitrogen, Carlsbad, CA, USA) according to the manufacturer’s instructions.

For stable gene silencing, two plasmids with shRNA (short hairpin RNA) targeting P2X7 were constructed using pGPU6/GFP/Neo cloning system (Genepharma Co, Ltd, Shanghai, China). The sequences targeting P2X7 were as follows: shRNA1, 5′-GCATGAATTATGGCACCATTA-3′, and shRNA2, 5′-GCAATTCAGGGCGGAATAATG-3′. The pGPU6/GFP/Neo vector with a scramble sequence shRNA was used as a negative control. Cell transfection was carried out using Lipofectamine 2000 (Invitrogen, Carlsbad, CA, USA) according to the manufacturer’s instructions. Cell clones with stable knockdown of P2X7 were established in 1E8 cells by Geneticin (G418) selection.

For gene over-expression, an expressing vector encoding full-length human P2X7 (hP2X7) was constructed (Genepharma Co, Ltd, Shanghai, China), and transfected into 22RV1 prostate cancer cells.

### In vitro migration assay and invasion assay

The migration and invasiveness abilities of prostate cancer cells were evaluated using Boyden Chamber assay according to the method described by Albini et al [28], with some modifications. Cell migration was analyzed by using 24-well Transwell chambers which contained 8 µM pore size polyethylene terephtalate membrane cell culture inserts (Costar, San Diego, CA, USA). The upper compartment was seeded with 0.5×10^5^ viable cells and the lower compartment was filled with 600 µl NIH3T3 conditioned medium as a chemoattractant. After incubation with or without ATP for 12 hours at 37°C in a humidified atmosphere containing 5% CO_2_, the chambers were removed. Cells on the upper side of the chamber were removed with cotton-tipped swabs, and cells on the underside of the membranes were stained with crystal violet after fixation in 4% formaldehyde. Cell invasive ability was assessed using the same inserts as mentioned above, but with the membrane covered with a film of Matrigel (BD, Franklin Lakes, NJ, USA). In this case, 1×10^5^ viable cells were seeded in the upper compartment. Membranes were cut and mounted onto slides and the cells were observed under the microscope at×200 magnification. Each experiment was repeated at least three times, and the results for migration and invasion were normalized to the controls.

### Ethics statement

All mice were raised in a specific pathogen free (SPF) environment. All the animals were handled according to the Guide for the Care and Use of Laboratory Animals. All experimental procedures and protocols were approved by the Institutional Animal Care and Use Committee of Peking University (No. LA2011-72).

### In vivo assay

The male BALB/c nude mice (4 weeks old) were purchased from Animal Department of Peking University Health Science Center and were randomized into three groups (n = 8 per group). Two 1E8 clones with stable knockdown of P2X7 as well as one negative control clone were used in the following experiments. Cells at the logarithmic growth phase were collected and adjusted with PBS to the concentration of 5×10^6^ cells/ml, and 200 µL cell suspension was injected subcutaneously into each anterior flank region of nude mice. Eight weeks after inoculation, all the mice were euthanized through cervical dislocation, and the primary tumors together with some organs such as livers, lungs, kidneys and lymph nodes were excised. Tumors, livers, lungs and kidneys of the mice were fixed in 4% paraformaldehyde, embedded in paraffin and then were sectioned into 4∼5 µm slices. To observe the distant micrometastasis of primary tumor, haematoxylin and eosin (HE) stain was performed on the tissues slides. Meanwhile, some tumor tissues were lysed with RIPA lysis buffer for detection of the protein expressions of EMT/invasion-related genes using western blot analysis. Furthermore, some tumor specimens were immersed in OTC medium, and frozen sections of 5 µm thickness were prepared for immunofluorescence assay.

### Immunofluorescence assay

For immunofluorescence staining, the tumor specimens were immersed in OTC medium, and frozen sections of 5 µm in thickness were prepared. Sections were fixed with acetone for 10 minutes at −20°C, and then nonspecific binding was blocked in goat serum for 1 h at room temperature. Sections were incubated overnight at 4°C with primary antibodies against Snail (Cell Signaling Technology), E-cadherin (Santa Cruz), or Claudin-1 (Invitrogen) respectively, and then incubated with a FITC-conjugated secondary antibody (Sigma) for 1 h at room temperature. DAPI staining was used to visualize nuclei. Images were taken with confocal microscopy.

### Fluo-4AM and ethidium bromide uptake assays

Changes in free internal calcium concentration were measured with the fluorescent indicator Fluo-4AM. Cells plated onto 1 mm glass bottom dish were loaded for 30 min at 37°C with 1 µM Fluo-4AM in HBSS buffer. Excess dye was removed by rinsing the cells twice with HBSS. Then, cells were incubated for an additional 10 min in HBSS, and treated with or without ATP and BzATP as indicated in figure legends. Images were acquired at 5 s intervals; numerous cells in a field of view were measured for 350 s to obtain the mean Fluo-4 fluorescent signal. To measure ethidium uptake, ethidium bromide (25 µM) was added to superfusion solutions. Images were acquired at 5 s intervals for 900 s. The total uptake of ethidium bromide was estimated by adding digitonin (100 µM) in the cuvette. The permeabilizing effect of ATP or BzATP was estimated by comparison with the permeabilization measured in the presence of digitonin.

### Cell survival assay

To assess the cell survival**,** 2×10^5^ cells were seeded per well in six-well plates in a control culture medium or in presence of 1 mM ATP (or 100 µM BzATP), and were grown for 24 h. Cell survival was estimated with MTT assay and data were expressed as % cell survival compared to control. Results were validated by manual cell counting. At least three independent experiments were performed.

### Data analysis and statistics

All experiments were repeated at least three times. Data are presented as means ± SD. The data were analyzed with SPSS 19.0. Student's *t*-test was performed to evaluate the differences between two groups, and nonparametric ANOVA was used when multiple means were compared. Differences were considered statistically significant at p<0.05.

## Results

### Prostate cancer cells expressed functional P2X7

**Figure 1:**
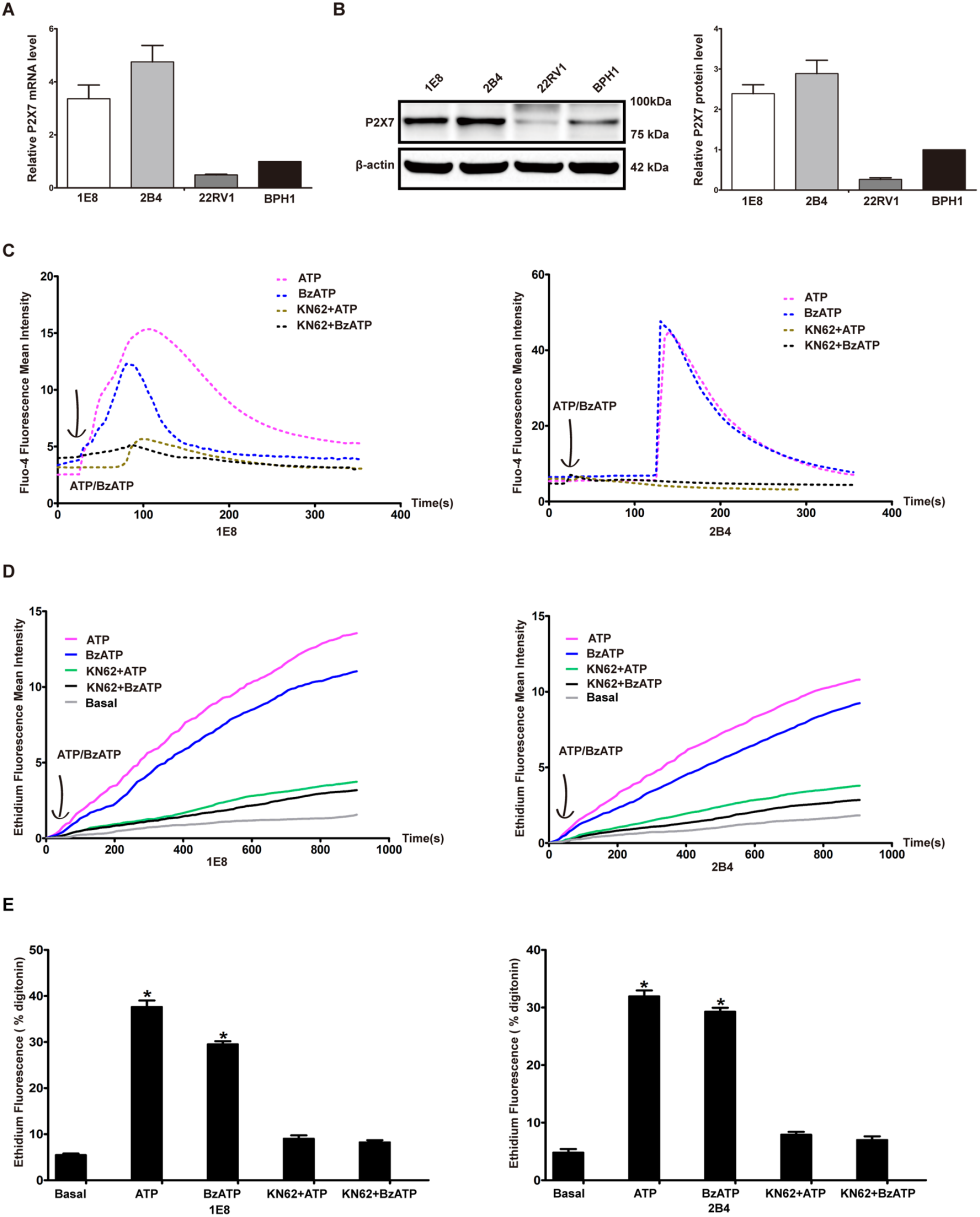
Prostate cancer cells expressed functional P2X7. (**A**) Relative mRNA expression of P2X7 was normalized by β-actin transcript in 1E8, 2B4, 22RV1 and BPH1 cells. (**B**) Protein levels of P2X7 in prostate cancer cells were detected by western blot analysis and data were normalized to β-actin. Protein expression of P2X7 in BPH1 cells was defined as 1. Values were presented as mean ± s.d. (vertical bars). (**C**) Representative intracellular calcium changes in response to ATP (1 mM) or BzATP (100 µM) in the presence or absence of KN62 (1 µM). (**D**) Representative ethidium uptake of prostate cancer cells in basal condition or upon stimulation with ATP (1 mM) or BzATP (100 µM) in the presence or absence of KN62. ATP/BzATP was added, as indicated by the arrow, to the HBSS containing 25 µM ethidium bromide. (**E**) Ethidium fluorescence intensity in basal condition or upon stimulation with ATP (1 mM) or BzATP (100 µM) in the presence or absence of KN62, was presented as a percentage relative to the value of digitonin-induced permeabilisation. At least three independent experiments were performed. *P<0.05.

Firstly, we analyzed the mRNA expression of P2X7 in prostate cancer cells 1E8, 2B4 and 22RV1 as well as in non-malignant immortalized prostate epithelial cell BPH1 using real-time PCR, and found that P2X7 was markedly expressed in 1E8 and 2B4 prostate cancer cells, while its expression was very faint in 22RV1 and BPH1 cells (Fig. 1A). In addition, P2X7 protein was highly expressed in 1E8 and 2B4 (Fig. 1B) prostate cancer cells. Next, we examined the intracellular free calcium concentration ([Ca^2+^]i) to determine whether P2X7 in prostate cancer cells was functional. Extracellular ATP (1 mM) or BzATP (100 µM) treatment triggered a remarkable increase of [Ca^2+^]i in prostate cancer cells, monitored by Fluo-4 fluorescence. However, KN62 (1 µM), a P2X7 antagonist, significantly inhibited ATP or BzATP induced [Ca^2+^]i increase (Fig. 1C). Ethidium uptake was also recorded in prostate cancer cells after stimulated with ATP or BzATP, which was significantly inhibited when KN62 was added to the external solution (Fig. 1D–E). All these results suggested that P2X7 expressed in prostate cancer cells was functional.

### P2X7 was involved in ATP/BzATP-driven migration and invasion of prostate cancer cells in vitro

**Figure 2:**
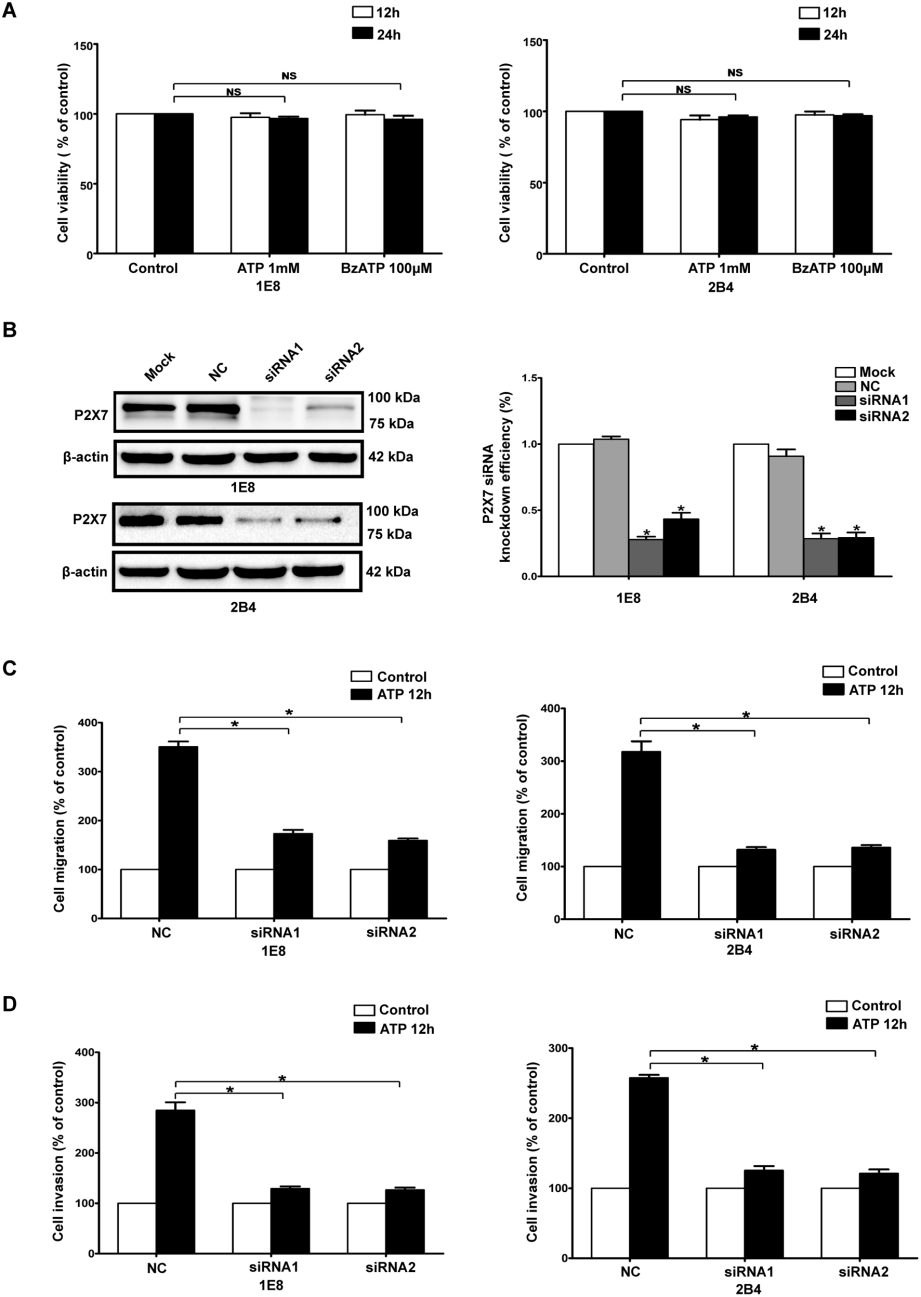
Knockdown of P2X7 attenuated ATP-driven migration and invasion in prostate cancer cells. (**A**) 1E8 and 2B4 prostate cancer cells were treated with 1 mM ATP for 12 h and 24 h. Cell survival was estimated by MTT assay and data were normalized to those under control condition. (**B**) 1E8 and 2B4 cells were transfected with two different P2X7 siRNAs (siRNA1 and siRNA2) or a control siRNA (NC). Western blot experiments were performed to detect the knockdown efficiency. (**C–D**) In vitro migration and invasion assays were carried out as described in methods section in the absence (Control) or presence of 1 mM ATP (ATP 12 h). Data of cell migration or invasion were calculated as a percentage of control cells. Results were demonstrated by histograms and values were presented as mean ± s.d. (vertical bars). At least three independent experiments were performed. *P<0.05.

P2X7 has been shown to be over-expressed in several tumors [15], [16], [17], [18], [19], however, its role in cancer progression remains unclear. Our previous studies demonstrated that extracellular ATP could enhance migration and invasion of prostate cancer cells [29], [30]. We wondered whether P2X7 played a role in the ATP-mediated biological behavior of prostate cancer cells. Firstly, we analyzed the effect of P2X7 on survival of prostate cancer cells, and found that activation of P2X7 with ATP or BzATP had no effect on cell viability (Fig. 2A). In contrast, we found that activation of P2X7 by ATP caused a significant enhancement of cell migration and invasion in prostate cancer cells (Fig. 2C–D). Then, two distinct siRNAs (siRNA1 and siRNA2) were used to silence P2X7 expression, and each siRNA achieved a prominent effect on knockdown of P2X7 in prostate cancer cells (Fig. 2B). Down-regulation of P2X7 by siRNA remarkably inhibited ATP-driven migration and invasion in 1E8 and 2B4 prostate cancer cells (Fig. 2C–D). Similarly, knockdown of P2X7 also significantly blocked BzATP-mediated migration and invasion of prostate cancer cells in 1E8 and 2B4 prostate cancer cells (S1 Figure). Furthermore, over-expression of P2X7 prominently enhanced ATP-induced migration and invasion in 22RV1 prostate cancer cells (S2A–B Figure). These results suggested that P2X7 was involved in the ATP-mediated migration and invasion of prostate cancer cells.

### P2X7 participated in the regulation of EMT/invasion-related gene expression in prostate cancer cells

**Figure 3:**
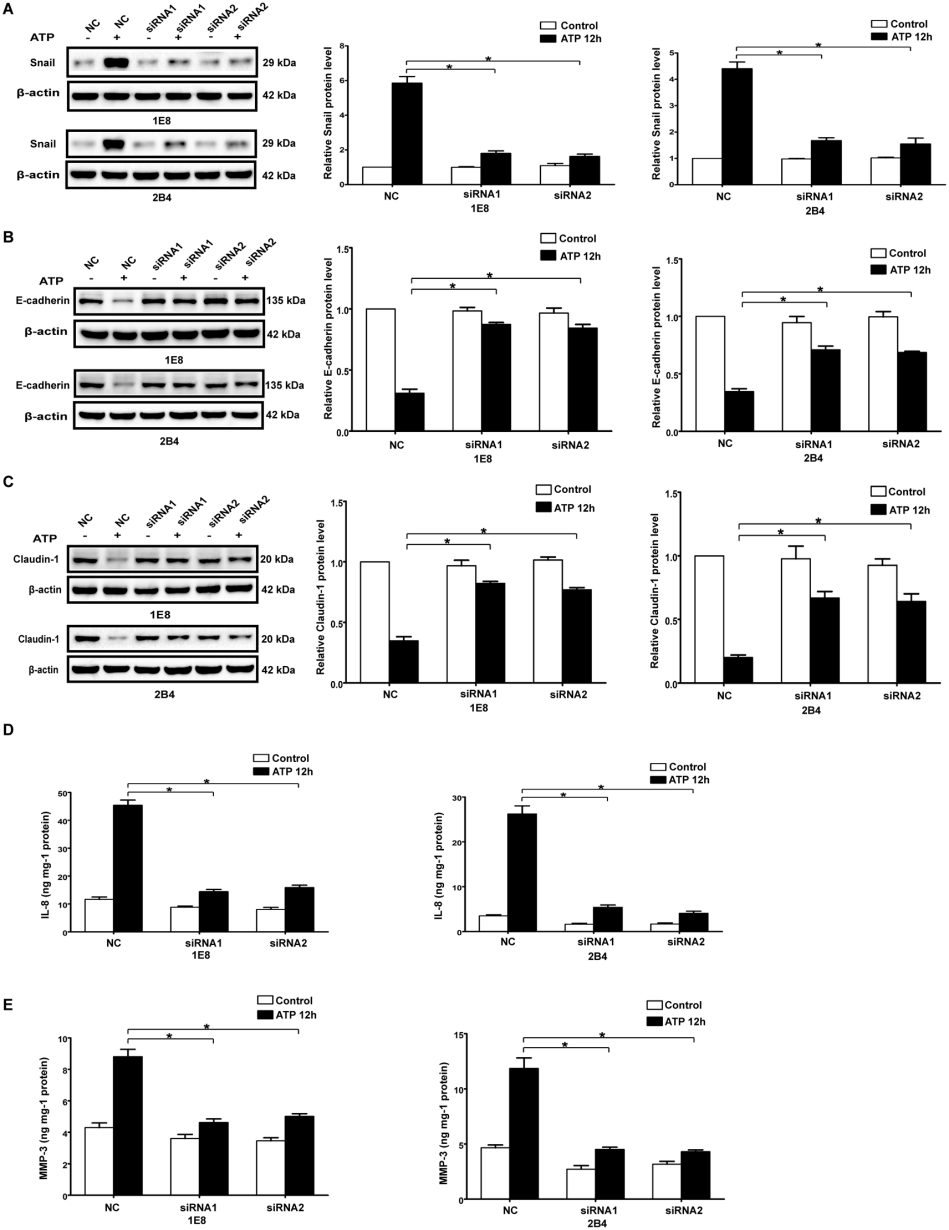
Knockdown of P2X7 attenuated ATP-mediated expression changes of EMT/invasion-related genes in prostate cancer cells. P2X7 silenced cells (siRNA1 and siRNA2) and control siRNA cells (NC) were treated with or without 1 mM ATP for 12 hours. Protein levels of Snail (**A**), E-cadherin (**B**) and Claudin-1 (**C**) were examined by Western blot analysis. Protein levels of IL-8 (**D**) and MMP-3 (**E**) were evaluated by ELISA assay. Expressions of these proteins were normalized to their respective expression in control cells (without ATP). Data were presented as mean ± s.d. (vertical bars). At least three independent experiments were performed. *P<0.05.

By using cDNA microarray analysis, our previous study revealed that extracellular ATP could regulate the mRNA expression of Snail, E-cadherin, Claudin-1 and IL-8, which play important roles in EMT and tumor progression [10]. Besides, our previous study in prostate cancer demonstrated that ATP treatment could increase the expression of MMP-3, which is extensively involved in invasion and metastasis of cancer [30]. Accordingly, we wondered whether P2X7 participated in these ATP-mediated gene expressions. As shown in Fig. 3, in the presence of ATP, expressions of Snail, IL-8 and MMP-3 were significantly increased in prostate cancer cells 1E8 and 2B4, while expressions of E-cadherin and Claudin-1 were prominently reduced. BzATP showed similar effect on the expressions of EMT/invasion-related genes in prostate cancer cells (S3 Figure). Nevertheless, after knockdown of P2X7 by siRNA, the changes in the expressions of Snail, IL-8, MMP-3, E-cadherin and Claudin-1, mediated by ATP or BzATP, were attenuated (Fig. 3 and S3 Figure). Moreover, specifically blocking the activation of P2X7 by its antagonist KN62, ATP and BzATP could not affect the expressions of Snail, E-cadherin and Claudin-1 anymore (S4 and S5 Figures). Similarly, ATP led to increased expression of Snail and decreased expression of E-cadherin in 22RV1 cells which exogenously expressed P2X7 (S2C Figure). All these data suggested that P2X7 was required for the ATP-mediated expression changes of EMT/invasion-related genes in prostate cancer cells.

### Activation of PI3K/AKT and ERK1/2 signaling pathways were critical in ATP−/BzATP-driven migration, invasion and expression changes of EMT/invasion-related genes

**Figure 4:**
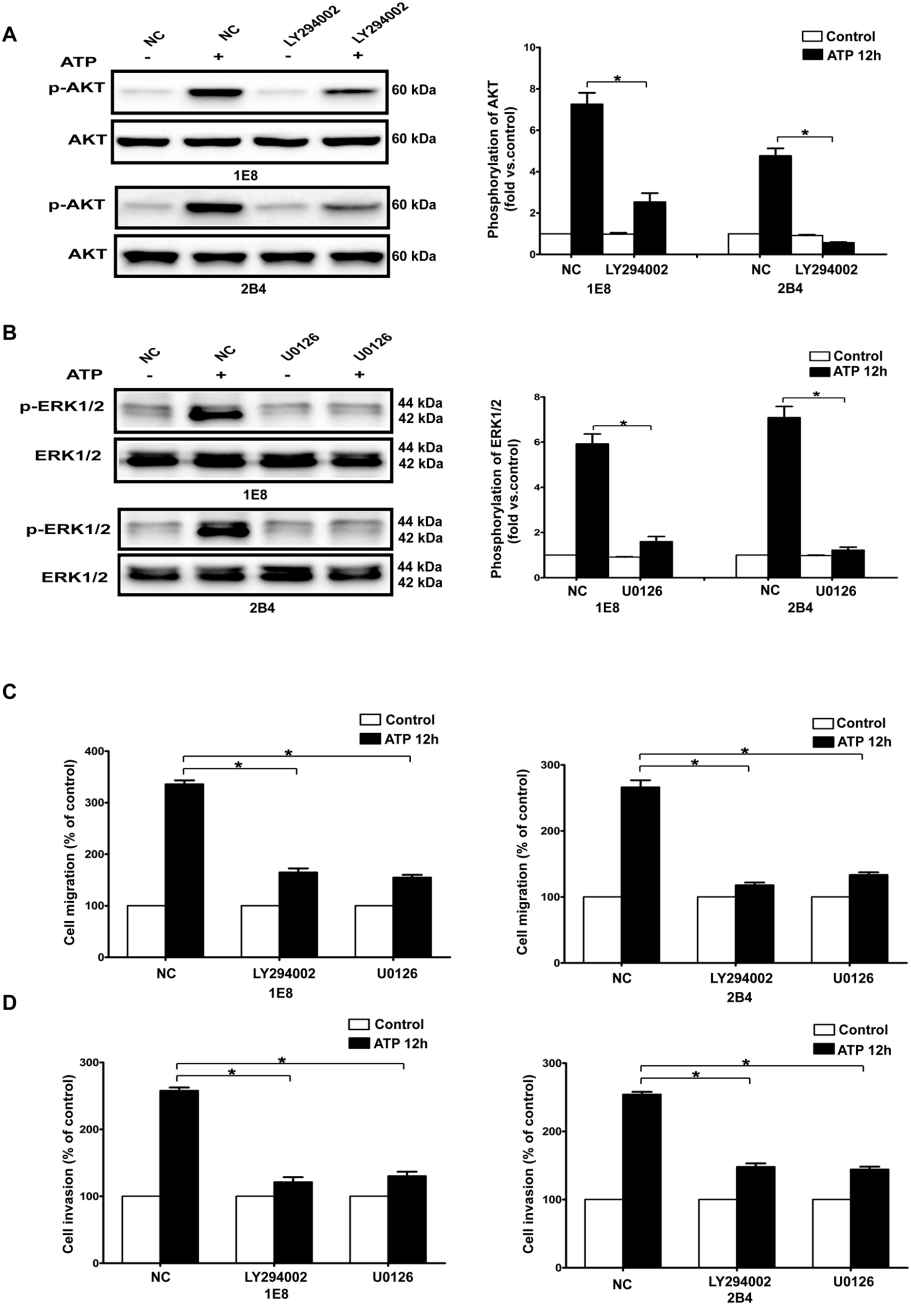
Effects of PI3K/AKT and ERK1/2 signaling pathways on ATP-mediated migration and invasion. IE8 and 2B4 cells were treated with LY294002 (lanes denoted as LY294002) or U0126 (lanes denoted as U0126) or without treatment (served as a negative control, lanes denoted as NC). (**A–B**) LY294002 and U0126 inhibited ATP-mediated PI3K/AKT and ERK1/2 activation respectively. (**C–D**) Effects of LY294002 and U0126 on migration and invasion in 1E8 and 2B4 prostate cancer cells. Data of cell migration or invasion were calculated as a percentage of control cells. Results were demonstrated by histograms, and values were presented as mean ± s.d. (vertical bars). At least three independent experiments were performed. *P<0.05.

**Figure 5:**
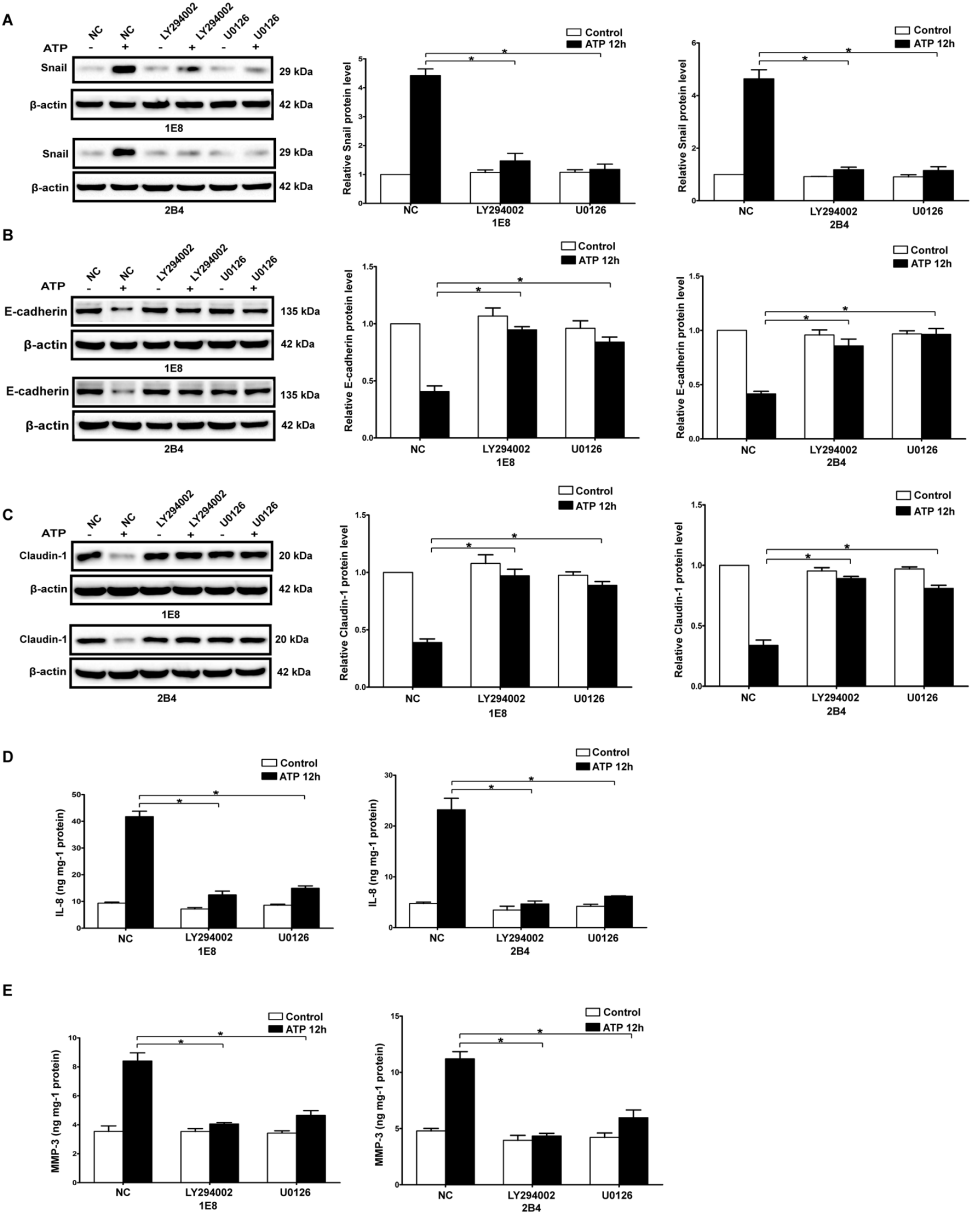
Effects of PI3K/AKT and ERK1/2 signaling pathways on ATP-induced expression changes of EMT/invasion-related genes. IE8 and 2B4 cells were treated with LY294002 (lanes denoted as LY294002) or U0126 (lanes denoted as U0126) or without treatment (served as a negative control, lanes denoted as NC). Expressions of Snail (**A**), E-cadherin (**B**) and Claudin-1 (**C**) were detected by western blots. Expressions of IL-8 (**D**) and MMP-3 (**E**) were detected using ELISA. Expressions of these proteins were normalized to their respective expression in control cells (without ATP). Data were presented as mean ± s.d. (vertical bars). At least three independent experiments were performed. *P<0.05.

In our previous study, it has been demonstrated that ATP activated PI3K/AKT and ERK1/2 signaling pathways in a time- and dose-dependent manner in 1E8 and 2B4 prostate cancer cells [29], [30]. Here, we found that BzATP treatment also caused a remarkable activation of PI3K/AKT and ERK1/2 signaling pathways in prostate cancer cells (S6A–B Figure). To understand the functional impact of PI3K/AKT and ERK1/2 signaling pathways in prostate cancer cells, two pharmacological inhibitors, LY294002 and U0126 were used to block PI3K/AKT and ERK1/2 signaling pathway respectively (Fig. 4A–B and S6A–B Figure). The results showed that ATP- and BzATP-driven migration and invasion of prostate cancer cells were significantly suppressed when PI3K/AKT or ERK1/2 signaling pathway was inhibited (Fig. 4C–D and S6C–D Figure). Furthermore, inhibition of either PI3K/AKT or ERK1/2 signaling pathway significantly attenuated the expression changes of EMT/invasion-related genes induced by ATP or BzATP in prostate cancer cells (Fig. 5 and S7 Figure).

### P2X7 was required for ATP/BzATP-induced activation of PI3K/AKT and ERK1/2 signaling pathways

**Figure 6:**
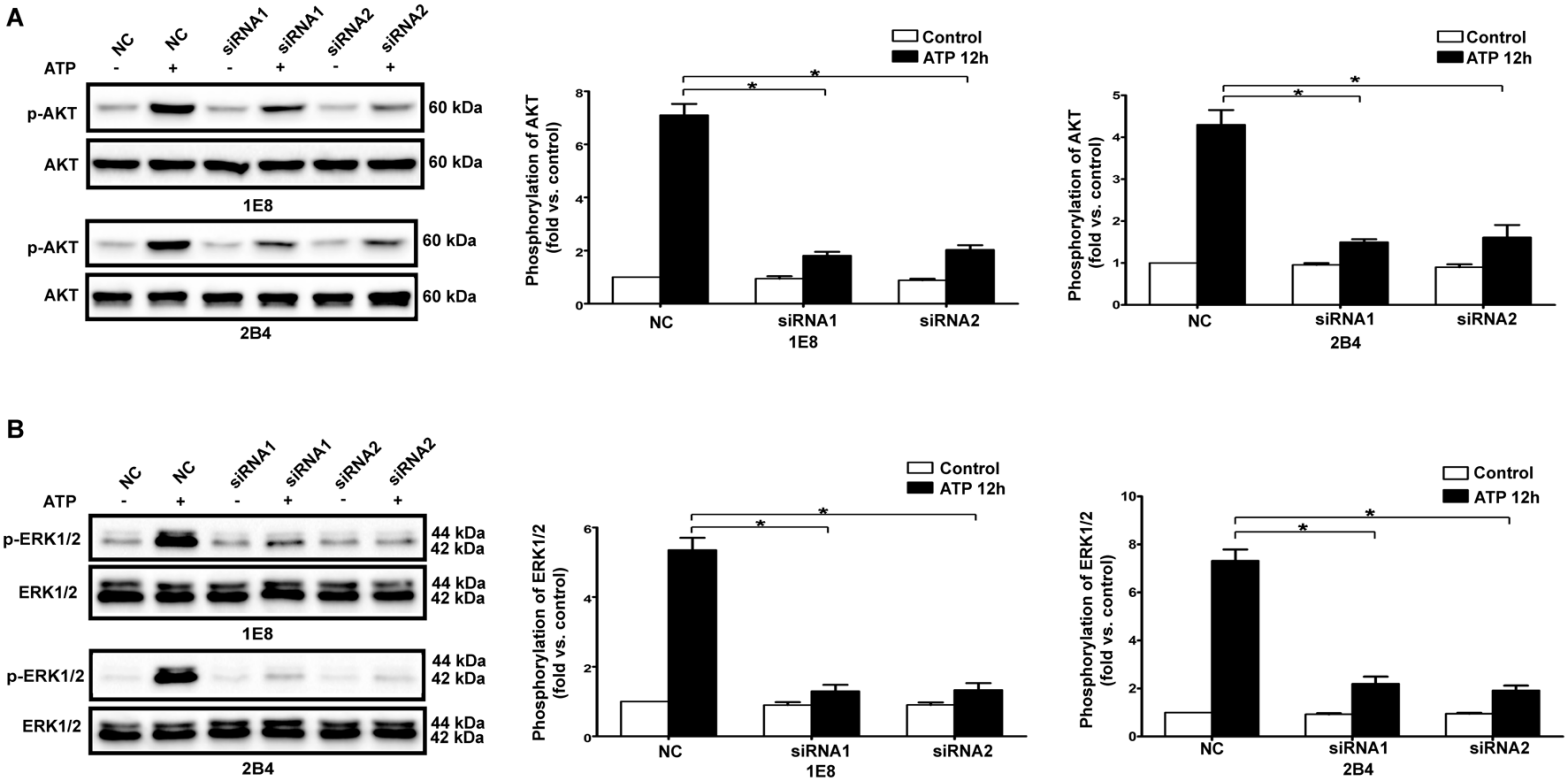
Knockdown of P2X7 attenuated ATP-mediated activation of PI3K/AKT and ERK1/2 signaling pathways. P2X7 silenced cells (siRNA1 and siRNA2) and control siRNA cells (NC) were treated with or without 1 mM ATP for 15 min. Western blot experiments were performed to analyze phosphorylation level of AKT (**A**) and ERK1/2 (**B**). Expressions of p-AKT and p-ERK1/2 were normalized to their respective expression in control cells (without ATP). Data were presented as mean ± s.d. (vertical bars). At least three independent experiments were performed. *P<0.05.

Since we observed that P2X7 as well as PI3K/AKT and ERK1/2 signaling pathways exhibited important effects on ATP- and BzATP-driven migration, invasion and expression changes of EMT/invasion-related genes in prostate cancer cells, we wondered whether P2X7 was involved in ATP- and BzATP-induced activation of PI3K/AKT and ERK1/2 signaling pathways. As shown in Fig. 6 and S8 Figure, knockdown of P2X7 resulted in prominent inhibition of ATP- and BzATP-induced phosphorylation of PI3K/AKT and ERK1/2 signaling pathways. Taken together, these results suggested an important role of P2X7 in ATP-mediated activation of PI3K/AKT and ERK1/2 signaling pathways.

### P2X7 was required for in vivo invasiveness and metastasis as well as regulation of the expression of EMT/invasion-related genes

**Figure 7:**
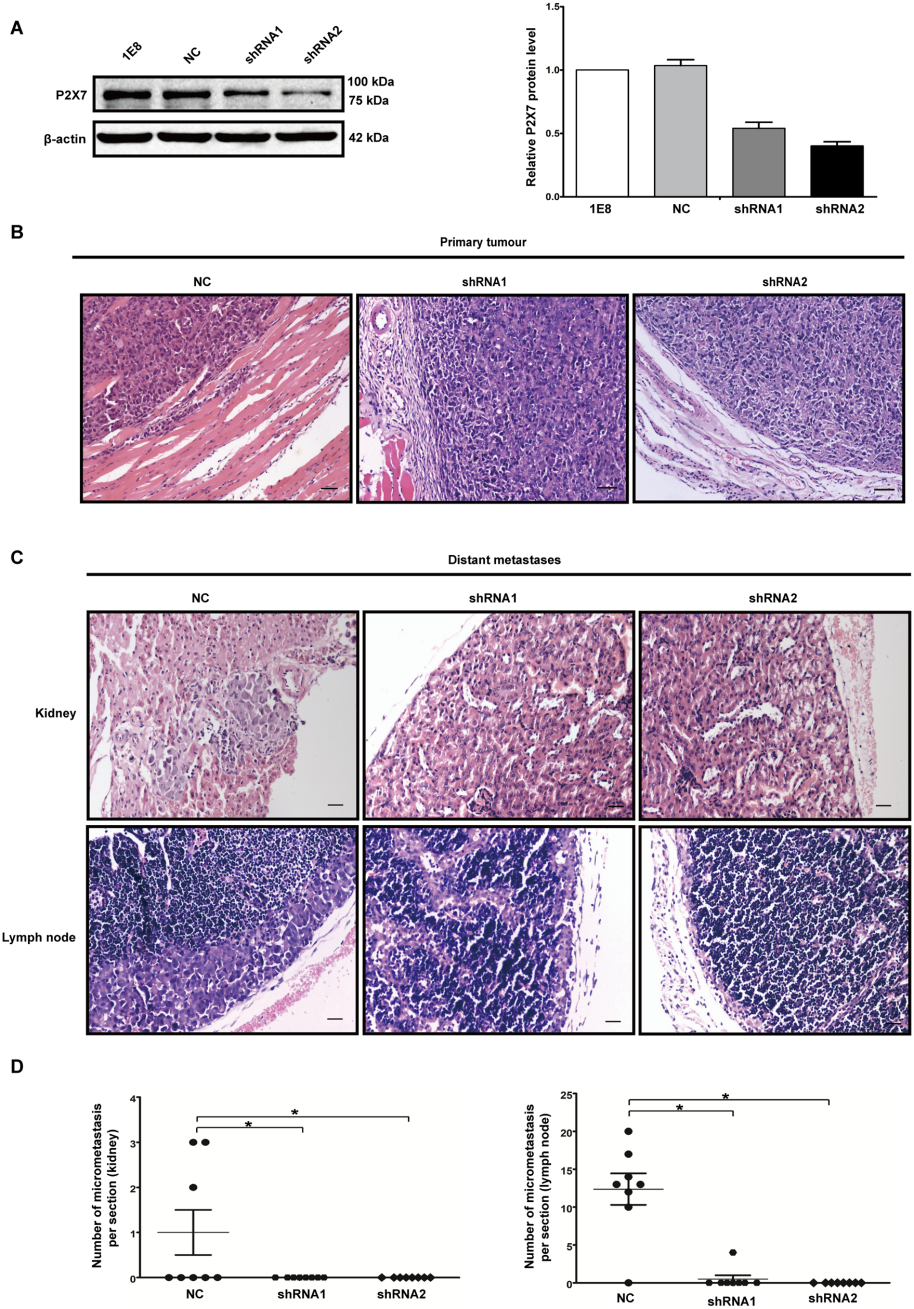
Knockdown of P2X7 suppressed invasiveness and metastases of prostate cancer cells in vivo. (**A**) 1E8 cells were stably transfected with P2X7 shRNA or a scramble shRNA (NC). Two stable P2X7 shRNA clones (shRNA1 and shRNA2) were shown to express low levels of P2X7 using western blot analysis. (**B**) Representative photograph of tumor sections and adjacent tissues (stained with haematoxylin and eosin (H&E)). Scale bars = 100 µm. (**C**) Representative photographs of kidney section and lymph node section (stained with H&E) from tumor-bearing mice. Scale bars = 50 µm. (**D**) The number of micrometastasis in kidney and lymph node per section from tumor-bearing mice. *P<0.05.

Finally, we analyzed the in vivo effect of P2X7 on invasion and metastases in nude mice. Tumors in mice, developed with subcutaneously injected control cells, displayed significant invasiveness to nearby tissues such as fat and muscle. Furthermore, in the mice injected with control cells, 37.5% of them presented distant metastases to kidney and 87.5% presented lymph node metastases. However, in the two groups injected with P2X7-silenced cells, only one mouse had metastasis to lymph node (Fig. 7).

**Figure 8:**
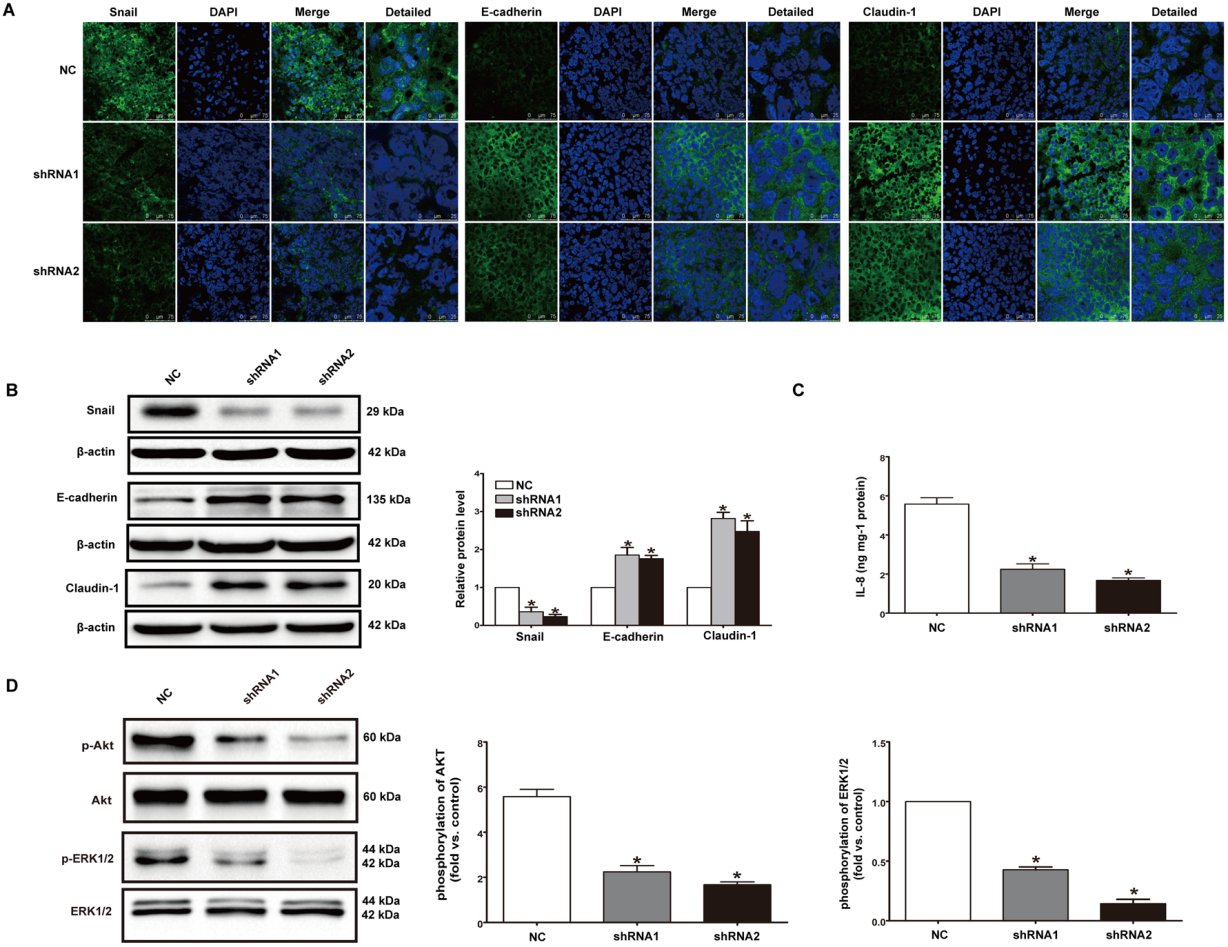
Knockdown of P2X7 affected expression of EMT/invasion-related genes as well as activation of PI3K/AKT and ERK1/2 signaling pathways in vivo. (**A**) Immunofluorescence staining for Snail, E-cadherin, Claudin-1 (green) and DAPI (blue) were performed in tumor tissues of mice. Images were taken by confocal microscopy. Scale bars represent 75 µm or 25 µm as shown in the Figure. (**B**) Western blotting was performed to detect the protein levels of Snail, E-cadherin and Claudin-1 in tumor tissues. (**C**) ELISA was used to examine the expression of IL-8. (**D**) Western blotting was performed to analyze the phosphorylation level of AKT and ERK1/2 in tumor tissues. For each group, at least three distinct tumors from three different mice were used in the experiments. *P<0.05.

We also analyzed the expressions of Snail, E-cadherin, Claudin-1 and IL-8 as well as the phosphorylation levels of AKT and ERK1/2 in primary tumor tissues of mice formed by 1E8 control shRNA cells and P2X7 shRNA cells. After knockdown of P2X7, expressions of Snail and IL-8 were obviously inhibited while expressions of E-cadherin and Claudin-1 were significantly increased (Fig. 8A–C). Besides, P2X7 knockdown significantly inhibited activation of AKT and ERK1/2 in tumor tissues (Fig. 8D). These results were consistent with the results observed in vitro.

## Discussion

Many studies demonstrated that the extracellular microenvironment of tumors contained much higher concentrations of ATP than healthy tissues, and ATP could represent a stressful stimulus in cancer progression [7]. ATP could be secreted by living tumor cells into their microenvironment at relatively high concentrations as well as be released from necrotic cells in the perilesional regions of cancers [6], [7], [24]. As a ubiquitous extracellular messenger, ATP functions via its interaction with P2X and P2Y receptors.

Among the P2 receptors engaged by extracellular ATP, P2X7 is the one most consistently expressed or even over-expressed by tumor cells. P2X7 is an ATP-gated ion channel and has long been known for its cytotoxic activity, however, increasing evidence suggested a role for P2X7 in cell proliferation. P2X7 has been demonstrated to stimulate proliferation of lymphoid cells and promote serum-independent growth [22], [23]. Extensive studies in immune cells showed the important role of P2X7 as an immunomodulatory receptor involved in interleukin (IL)-1β maturation and release, antigen presentation and graft-versus-host reaction [31], [32], [33]. Over the last decade, high-expression of P2X7 has been found in diverse kinds of tumors and evidence has accumulated showing the pro-cancerous effects of P2X7 [15], [16], [17]. Melanoma cells with highly expressed P2X7, displayed anti-apoptotic effect [16]. After transfection with P2X7, HEK293 fibroblasts and CT26 colon carcinoma cells demonstrated with enhancements in tumorigenesis, in vivo growth and angiogenesis, and reduction in apoptosis [34]. Besides, activation of P2X7 promoted migration and invasiveness of breast cancer cells [24] and lymphoid neoplasm cells [35]. In the present study, we demonstrated that P2X7 was highly expressed in some prostate cancer cells. Knockdown of P2X7 by siRNA significantly abrogated ATP-enhanced migration and invasion in vitro. Besides, down-regulation of P2X7 led to remarkable inhibition of tumor invasiveness and metastases in nude mice. Furthermore, over-expression of P2X7 markedly enhanced ATP-mediated migration and invasion in 22RV1 prostate cancer cells, providing further evidence that P2X7 was one of the key regulators of prostate cancer invasion and metastases.

The epithelial-mesenchymal transition (EMT) is an important step during cancer progression and closely related to high motile and invasive characteristic of cancer cells. It is now widely accepted that impaired expression or function of E-cadherin is one of the earliest steps in EMT [3]. In addition to adhesion molecules, during the progression of EMT, tight junction proteins, such as Claudins, are usually down-regulated [36]. Claudins play a crucial role in the maintenance of cell polarity and have a significant influence on tumor progression in several types of cancers [37], [38], [39]. Furthermore, several transcription factors such as Snail, Slug and Twist have been reported to drive EMT in various cancer cells, and Snail, a major transcription factor, is generally up-regulated during the EMT process [3]. In the present study, we demonstrated that ATP and BzATP treatment both led to a significant up-regulation of Snail as well as a dramatic down-regulation of E-cadherin and Claudin-1 in prostate cancer cells in vitro, and P2X7 knockdown remarkably inhibited expression of Snail and promoted expressions of E-cadherin and Claudin-1 in vitro and in vivo. These results suggested that P2X7 mediated the ATP-driven EMT in prostate cancer cells.

Tumor progression and metastasis do not only depend upon the malignant potential of cancer cells themselves, but also the influence of various regulatory factors in tumor environment. It has been reported that IL-8 functions as a significant regulatory factor in tumor microenvironment and exerts profound effects on tumor angiogenesis, proliferation, invasion and metastasis in several cancer cells, including prostate cancer cells [40], [41], [42], Furthermore, clinical studies on advanced stages of prostate cancer cases showed that nonapical and cytoplasmic expression of interleukin-8 correlated with tumor proliferation and microvessel density [43]. In the present study, expression of IL-8 was strikingly increased after ATP treatment in prostate cancer cells, and this expression change was significantly attenuated both in vitro and in vivo after P2X7 knockdown, suggesting a critical role of P2X7 in the ATP-mediated up-regulation of IL-8.

As Martin illustrated [44], tumor cells acquire some malignant properties to invade surrounding tissues and metastasize to distant site are reflective of aberrant signaling pathways. PI3K/AKT and ERK1/2 signaling pathways are critical signaling pathways that are closely related to a variety of tumor-promoting activities such as cell proliferation, migration and angiogenesis. Our previous researches have shown that ATP could activate PI3K/AKT and ERK1/2 signaling pathways [29], [30]. Here, it was revealed that ATP-driven migration, invasion and expression changes of EMT/invasion-related genes were dependent on P2X7-mediated activation of PI3K/AKT and ERK1/2 signaling pathways. These data suggest that P2X7 probably functions through activation of PI3K/AKT and ERK1/2 signaling pathways to mediate ATP-driven prostate cancer progression.

In summary, our studies in vitro strongly suggested that P2X7 played an important role in ATP-induced expression changes of EMT/invasion-related markers and subsequent enhancement of migration and invasiveness of prostate cancer cells, and P2X7 was required for ATP-mediated activation of PI3K/AKT and ERK1/2 signaling pathways. Besides, the in vivo experiments demonstrated a novel oncogenic role of P2X7 in prostate cancer spreading. P2X7 could be considered as a promising therapeutic target of prostate cancer.

It must be pointed out that both P2X7 and P2Y2 [10] receptors showed important roles in the ATP-promoted invasion and metastasis of prostate cancer in our studies. However, the elaborate interaction between them and how they function individually or cooperatively in vivo when they both present in the cells remains largely unknown. Double silencing strategy may help explain some of the mechanisms. Besides, the partial action of KN62 at the kinetics of calcium entry and permeabilisation seems to indicate that some other purinergic receptor subtypes could be involved in ATP-mediated progression of prostate cancer. Thus, the detailed molecular mechanisms of purinergic signaling in the progression of prostate cancer need to be clarified in the future.

## Supporting Information

S1 Figure **Down-regulation of P2X7 attenuated BzATP-driven migration and invasion in prostate cancer cells.** 1E8 and 2B4 prostate cancer cells were transfected with two different P2X7 siRNAs (siRNA1 and siRNA2) or a control siRNA (NC). Cell migration and invasion assays were carried out as described in methods section in the absence (Control) or presence of 100 µM BzATP (BzATP 12 h). Data of cell migration **(A)** or invasion **(B)** were calculated as a percentage of control cells. Results were demonstrated by histograms and values were presented as mean ± s.d. (vertical bars). At least three independent experiments were performed. *P<0.05.
(TIF)

S2 Figure **Extracellular ATP regulated migration, invasion and expression of EMT-related genes in 22RV1 prostate cancer cells after over-expression of P2X7.** 22RV1 prostate cancer cells were transfected with an expressing vector encoding full-length human P2X7 (denoted as hP2X7) or an empty vector (denoted as NC). **(A)** Western blot was performed to evaluate the over-expression efficiency of P2X7. **(B)** Over-expression of P2X7 significantly enhanced ATP-mediated migration and invasion in 22RV1 prostate cancer cells. **(C)** Western blot experiments were carried out to detect protein levels of Snail and E-cadherin. Expressions of Snail and E-cadherin were normalized to their respective expression in control cells. Data were presented as mean ± s.d. (vertical bars). At least three independent experiments were performed. *P<0.05.
(TIF)

S3 Figure **Knockdown of P2X7 attenuated BzATP-mediated expression changes of EMT/invasion-related genes in prostate cancer cells.** P2X7 silenced cells (siRNA1 and siRNA2) and control siRNA cells (NC) were treated with or without 100 µM BzATP for 12 hours. Protein levels of Snail **(A)**, E-cadherin **(B)** and Claudin-1 **(C)** were examined by Western blot analysis. Protein levels of IL-8 **(D)** and MMP-3 **(E)** were evaluated by ELISA assay. Expressions of Snail, E-cadherin, Claudin-1, IL-8 and MMP-3 were normalized to their respective expression in control cells (without BzATP). Data were presented as mean ± s.d. (vertical bars). At least three independent experiments were performed. *P<0.05.
(TIF)

S4 Figure **ATP-induced EMT was P2X7 dependent in prostate cancer cells.** 1E8 and 2B4 prostate cancer cells were treated with 1 mM ATP in the presence or absence of KN62 for 12 h. Western blot experiments were performed to examine protein levels of Snail **(A)**, E-cadherin **(B)** and Claudin-1. **(C)** Expressions of these proteins were normalized to their respective expression in control cells (without ATP). Data were presented as mean ± s.d. (vertical bars). At least three independent experiments were performed. *P<0.05.
(TIF)

S5 Figure **P2X7 was required for BzATP-mediated EMT in prostate cancer cells.** 1E8 and 2B4 prostate cancer cells were treated with 100 µM BzATP in the presence or absence of KN62 for 12 h. Western blot experiments were performed to examine protein levels of Snail **(A)**, E-cadherin **(B)** and Claudin-1. **(C)** Expressions of these proteins were normalized to their respective expression in control cells (without BzATP). Data were presented as mean ± s.d. (vertical bars). At least three independent experiments were performed. *P<0.05.
(TIF)

S6 Figure **Effects of PI3K/AKT and ERK1/2 signaling pathways on BzATP-mediated migration and invasion.** IE8 and 2B4 cells were treated with LY294002 (lanes denoted as LY294002) or U0126 (lanes denoted as U0126) or without treatment (served as a negative control, lanes denoted as NC). **(A–B)** LY294002 and U0126 inhibited BzATP-mediated PI3K/AKT and ERK1/2 activation respectively. **(C–D)** Effects of LY294002 and U0126 on migration and invasion in 1E8 and 2B4 prostate cancer cells. Data were calculated as a percentage of control cells. Values were presented as mean ± s.d. (vertical bars). At least three independent experiments were performed. *P<0.05.
(TIF)

S7 Figure **Effects of PI3K/AKT and ERK1/2 signaling pathways on BzATP-induced expression changes of EMT/invasion-related genes.** IE8 and 2B4 cells were treated with LY294002 (lanes denoted as LY294002) or U0126 (lanes denoted as U0126) or without treatment (served as a negative control, lanes denoted as NC). Expressions of Snail **(A)**, E-cadherin **(B)** and Claudin-1 **(C)** were detected by western blots. Expression of IL-8 **(D)** and MMP-3 **(E)** were detected using ELISA. Expressions of these proteins were normalized to their respective expression in control cells (without BzATP). Data were presented as mean ± s.d. (vertical bars). At least three independent experiments were performed. *P<0.05.
(TIF)

S8 Figure **Knockdown of P2X7 attenuated BzATP-mediated activation of PI3K/AKT and ERK1/2 signaling pathways.** P2X7 silenced cells (siRNA1 and siRNA2) and control siRNA cells (NC) were treated with or without 100 µM BzATP for 15 min. Western blot experiments were performed to analyze phosphorylation level of AKT **(A)** and ERK1/2 **(B)**. Expression of p-AKT and p-ERK1/2 were normalized to their respective expression in control cells (without BzATP). Data were presented as mean ± s.d. (vertical bars). At least three independent experiments were performed. *P<0.05.
(TIF)

S1 Table **Sequence of the primers used in the real-time qPCR experiments.**
(DOC)
