# Correction: P2X7 Mediates ATP-Driven Invasiveness in Prostate Cancer Cells

**DOI:** 10.1371/journal.pone.0123388

**Published:** 2015-04-07

**Authors:** The PLOS ONE Staff

There is an error in Fig. 3. The protein band of Caludin-1 in Fig. 3C is incorrect. The authors have provided a corrected version here.

**Fig 3:**
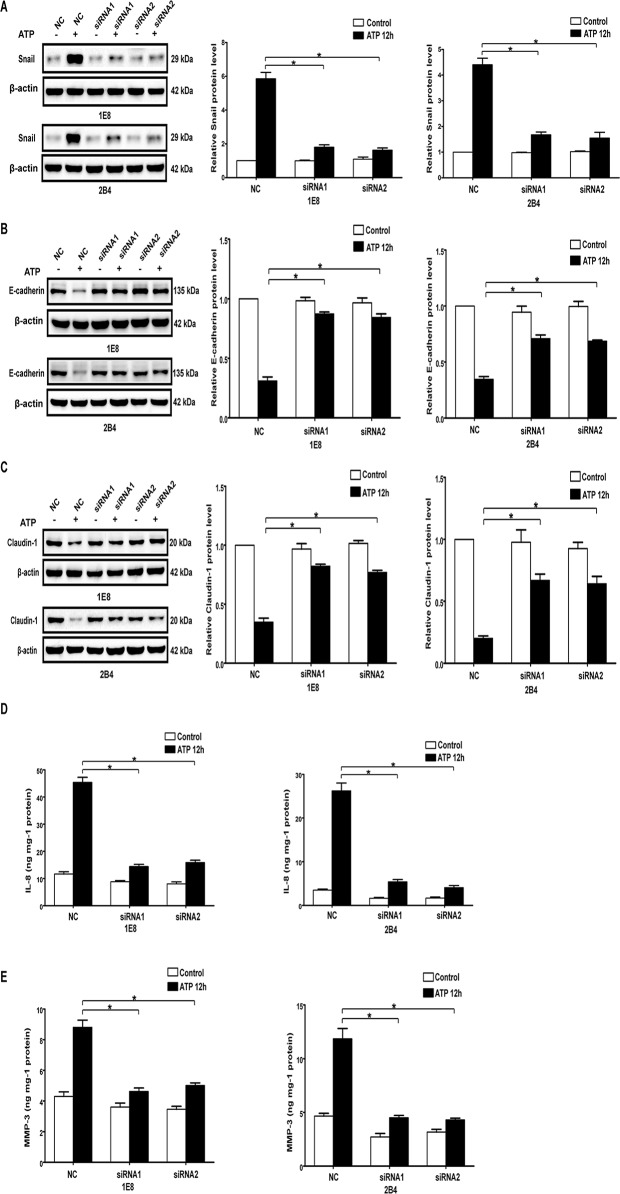
Knockdown of P2X7 attenuated ATP-mediated expression changes of EMT/invasion-related genes in prostate cancer cells. P2X7 silenced cells (siRNA1 and siRNA2) and control siRNA cells (NC) were treated with or without 1 mM ATP for 12 hours. Protein levels of Snail (A), E-cadherin (B) and Claudin-1 (C) were examined by Western blot analysis. Protein levels of IL-8 (D) and MMP-3 (E) were evaluated by ELISA assay. Expressions of these proteins were normalized to their respective expression in control cells (without ATP). Data were presented as mean ± s.d. (vertical bars). At least three independent experiments were performed. *P<0.05.

There is an error in Fig. 8. The phsosphorylation of AKT histogram in Fig. 8D is incorrect. The authors have provided a corrected version here.

**Fig 8:**
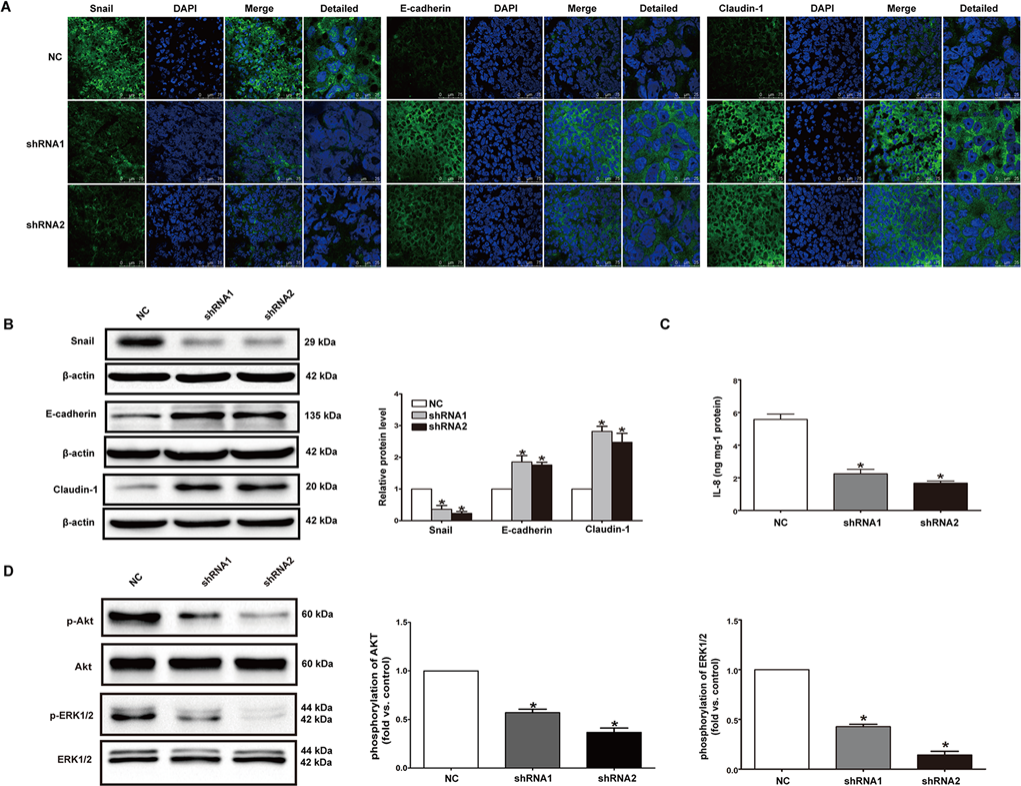
Knockdown of P2X7 affected expression of EMT/invasion-related genes as well as activation of PI3K/AKT and ERK1/2 signaling pathways in vivo. (**A**) Immunofluorescence staining for Snail, E-cadherin, Claudin-1 (green) and DAPI (blue) were performed in tumor tissues of mice. Images were taken by confocal microscopy. Scale bars represent 75 μm or 25 μm as shown in the Figure. (**B**) Western blotting was performed to detect the protein levels of Snail, E-cadherin and Claudin-1 in tumor tissues. (**C**) ELISA was used to examine the expression of IL-8. (**D**) Western blotting was performed to analyze the phosphorylation level of AKT and ERK1/2 in tumor tissues. For each group, at least three distinct tumors from three different mice were used in the experiments. *P<0.05.
