# Supplementary figures and images for: P2X7 Mediates ATP-Driven Invasiveness in Prostate Cancer Cells
Source: PLoS One. 2014 Dec 8;9(12):e114371. doi: 10.1371/journal.pone.0114371 (PMC4259308; doi:10.1371/journal.pone.0114371)

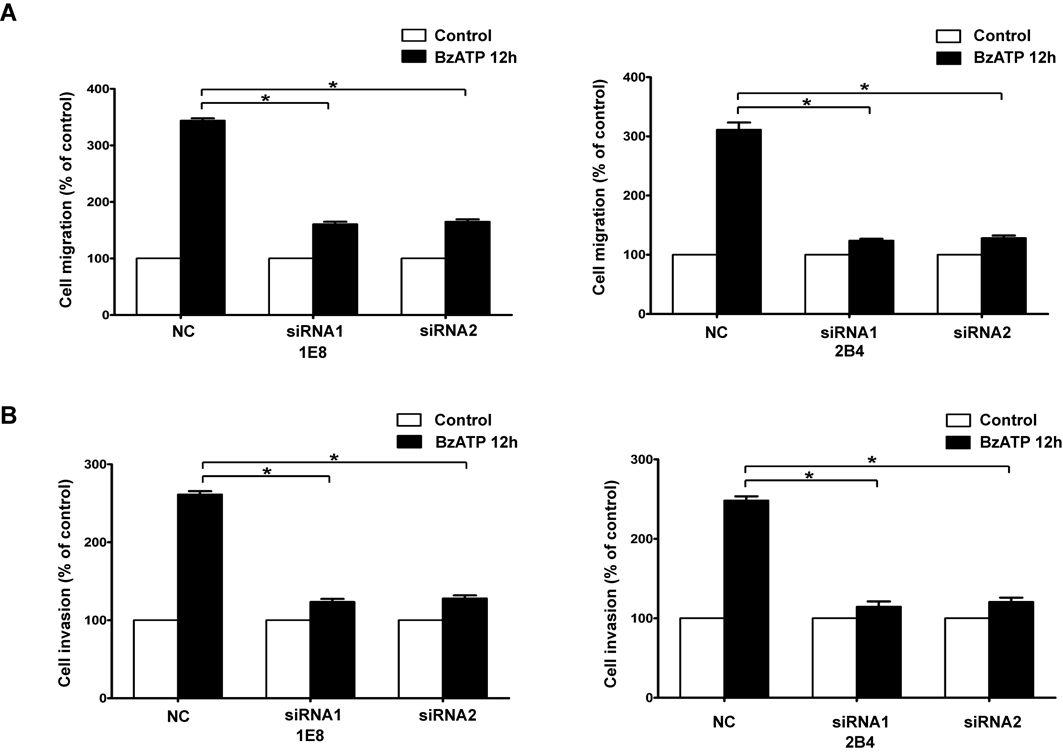

Supplement: S1 Figure — Down-regulation of P2X7 attenuated BzATP-driven migration and invasion in prostate cancer cells. 1E8 and 2B4 prostate cancer cells were transfected with two different P2X7 siRNAs (siRNA1 and siRNA2) or a control siRNA (NC). Cell migration and invasion assays were carried out as described in methods section in the absence (Control) or presence of 100 µM BzATP (BzATP 12 h). Data of cell migration (A) or invasion (B) were calculated as a percentage of control cells. Results were demonstrated by histograms and values were presented as mean ± s.d. (vertical bars). At least three independent experiments were performed. *P<0.05. (TIF) [file pone.0114371.s001.tif]

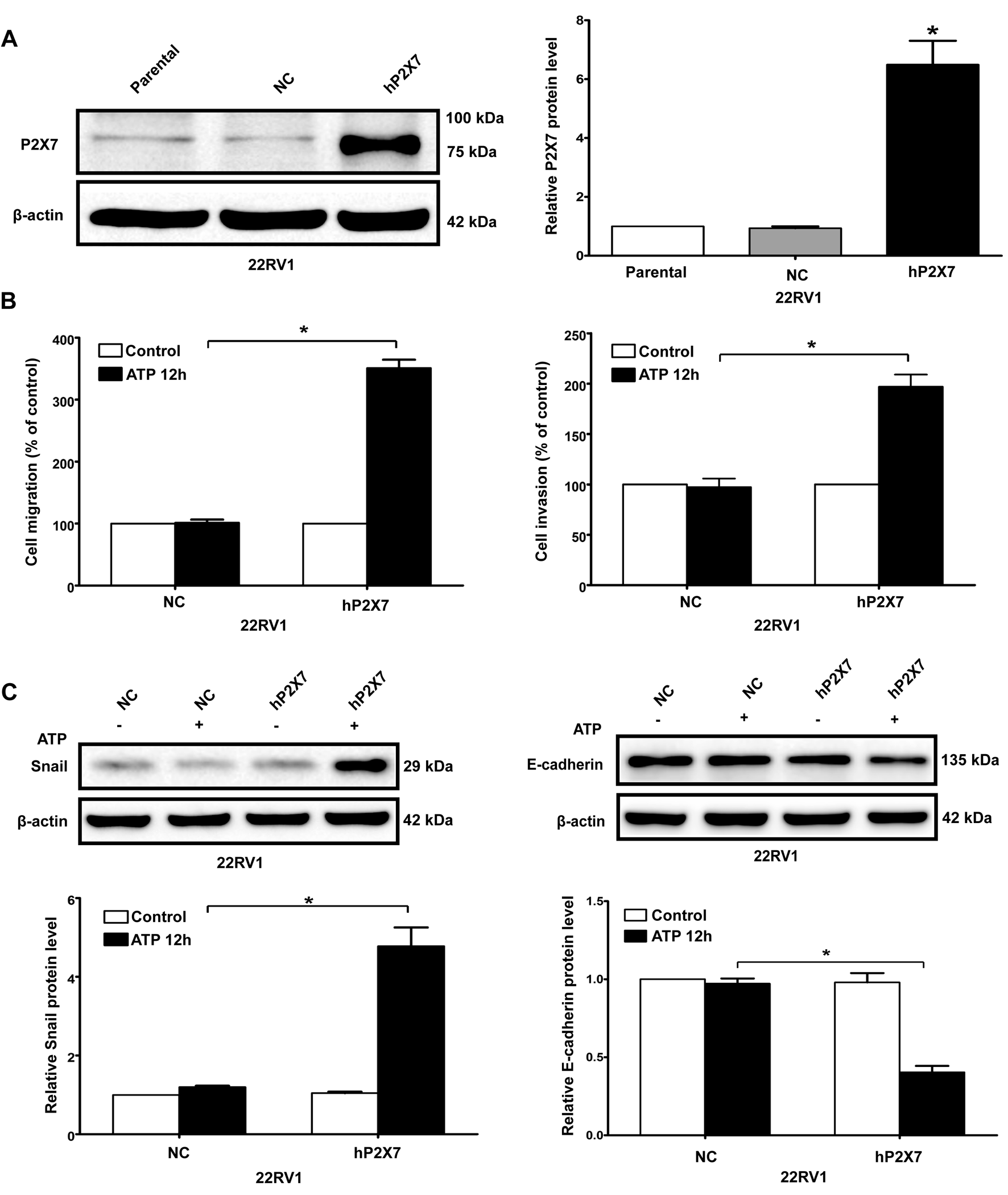

Supplement: S2 Figure — Extracellular ATP regulated migration, invasion and expression of EMT-related genes in 22RV1 prostate cancer cells after over-expression of P2X7. 22RV1 prostate cancer cells were transfected with an expressing vector encoding full-length human P2X7 (denoted as hP2X7) or an empty vector (denoted as NC). (A) Western blot was performed to evaluate the over-expression efficiency of P2X7. (B) Over-expression of P2X7 significantly enhanced ATP-mediated migration and invasion in 22RV1 prostate cancer cells. (C) Western blot experiments were carried out to detect protein levels of Snail and E-cadherin. Expressions of Snail and E-cadherin were normalized to their respective expression in control cells. Data were presented as mean ± s.d. (vertical bars). At least three independent experiments were performed. *P<0.05. (TIF) [file pone.0114371.s002.tif]

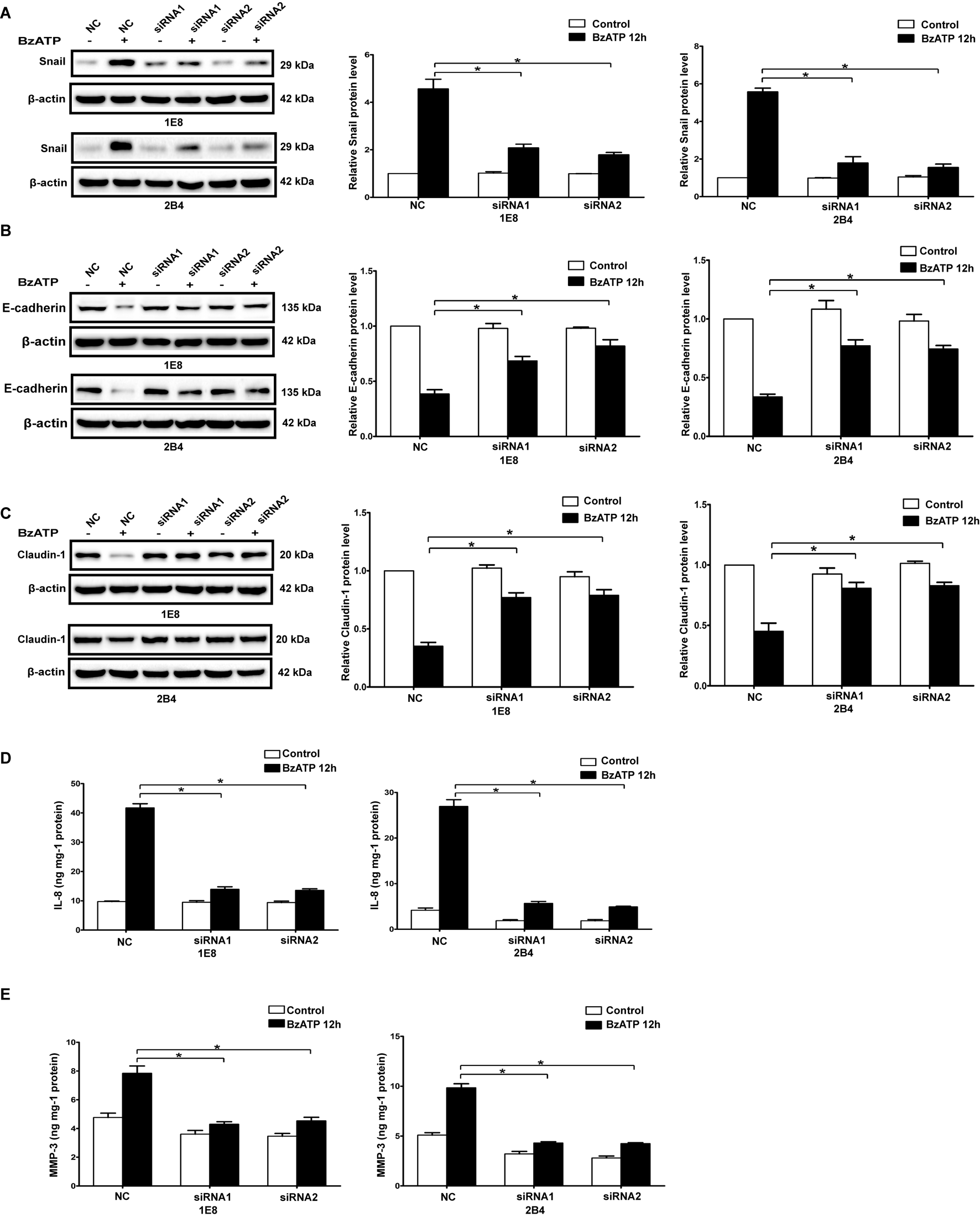

Supplement: S3 Figure — Knockdown of P2X7 attenuated BzATP-mediated expression changes of EMT/invasion-related genes in prostate cancer cells. P2X7 silenced cells (siRNA1 and siRNA2) and control siRNA cells (NC) were treated with or without 100 µM BzATP for 12 hours. Protein levels of Snail (A), E-cadherin (B) and Claudin-1 (C) were examined by Western blot analysis. Protein levels of IL-8 (D) and MMP-3 (E) were evaluated by ELISA assay. Expressions of Snail, E-cadherin, Claudin-1, IL-8 and MMP-3 were normalized to their respective expression in control cells (without BzATP). Data were presented as mean ± s.d. (vertical bars). At least three independent experiments were performed. *P<0.05. (TIF) [file pone.0114371.s003.tif]

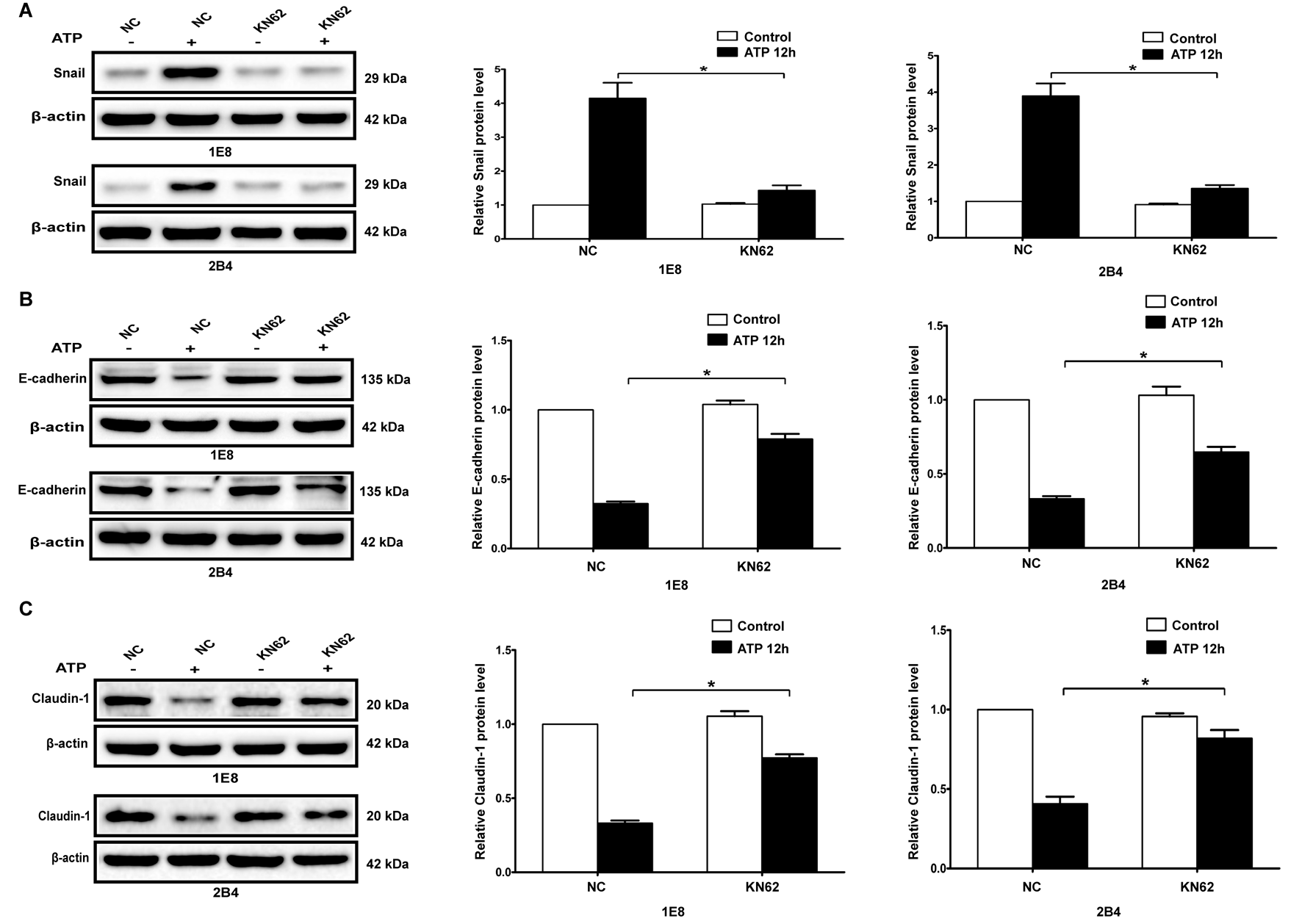

Supplement: S4 Figure — ATP-induced EMT was P2X7 dependent in prostate cancer cells. 1E8 and 2B4 prostate cancer cells were treated with 1 mM ATP in the presence or absence of KN62 for 12 h. Western blot experiments were performed to examine protein levels of Snail (A), E-cadherin (B) and Claudin-1. (C) Expressions of these proteins were normalized to their respective expression in control cells (without ATP). Data were presented as mean ± s.d. (vertical bars). At least three independent experiments were performed. *P<0.05. (TIF) [file pone.0114371.s004.tif]

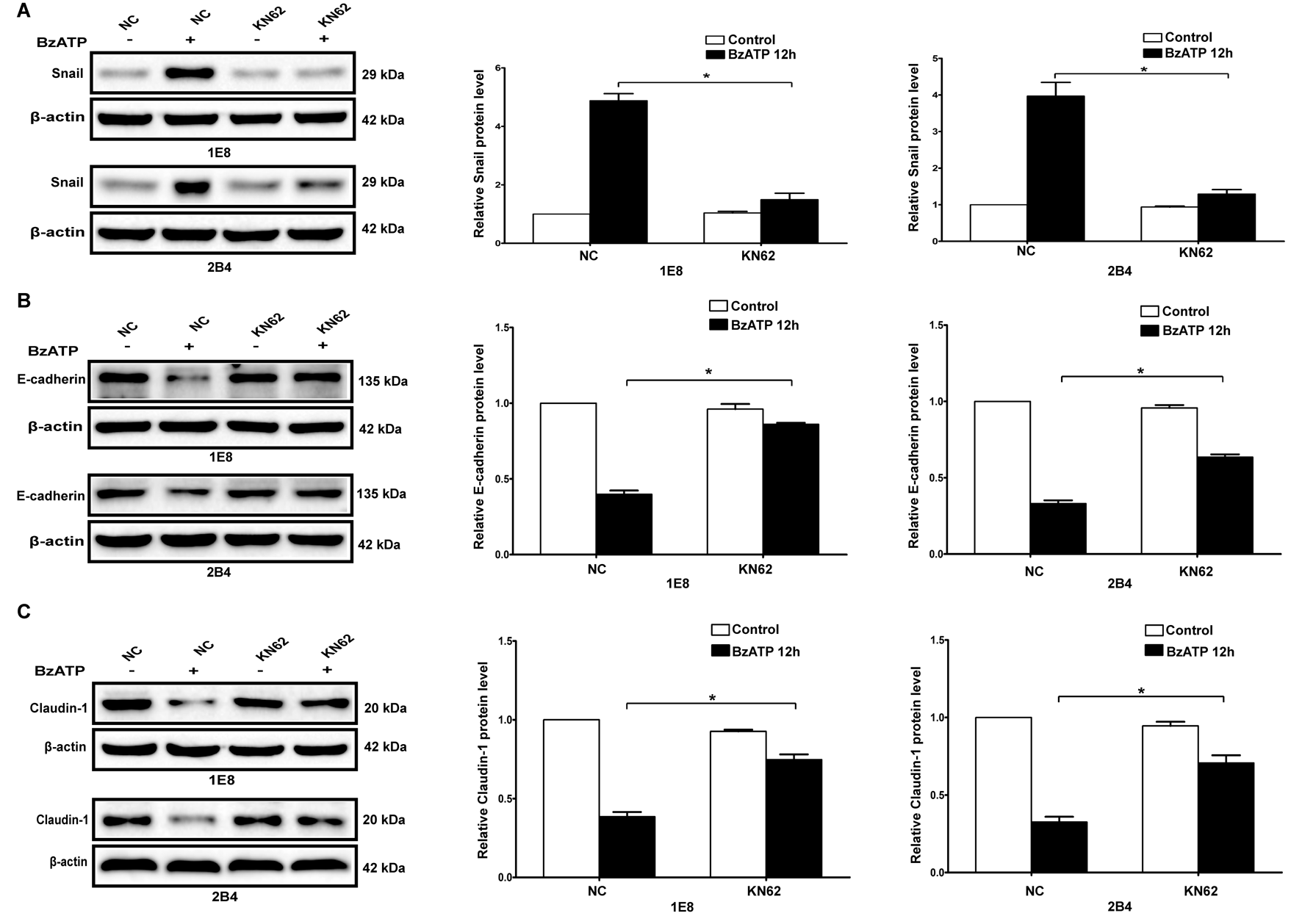

Supplement: S5 Figure — P2X7 was required for BzATP-mediated EMT in prostate cancer cells. 1E8 and 2B4 prostate cancer cells were treated with 100 µM BzATP in the presence or absence of KN62 for 12 h. Western blot experiments were performed to examine protein levels of Snail (A), E-cadherin (B) and Claudin-1. (C) Expressions of these proteins were normalized to their respective expression in control cells (without BzATP). Data were presented as mean ± s.d. (vertical bars). At least three independent experiments were performed. *P<0.05. (TIF) [file pone.0114371.s005.tif]

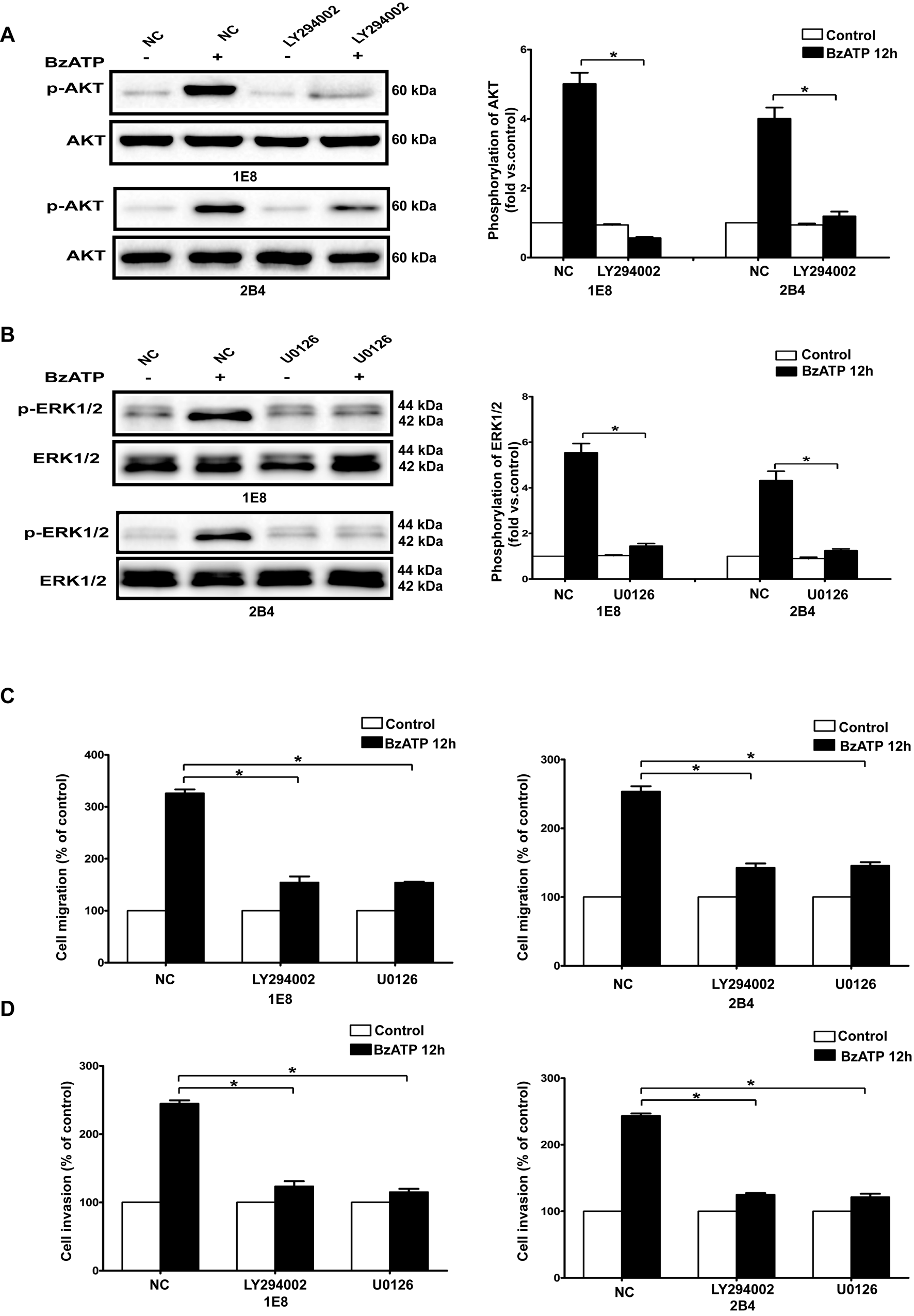

Supplement: S6 Figure — Effects of PI3K/AKT and ERK1/2 signaling pathways on BzATP-mediated migration and invasion. IE8 and 2B4 cells were treated with LY294002 (lanes denoted as LY294002) or U0126 (lanes denoted as U0126) or without treatment (served as a negative control, lanes denoted as NC). (A–B) LY294002 and U0126 inhibited BzATP-mediated PI3K/AKT and ERK1/2 activation respectively. (C–D) Effects of LY294002 and U0126 on migration and invasion in 1E8 and 2B4 prostate cancer cells. Data were calculated as a percentage of control cells. Values were presented as mean ± s.d. (vertical bars). At least three independent experiments were performed. *P<0.05. (TIF) [file pone.0114371.s006.tif]

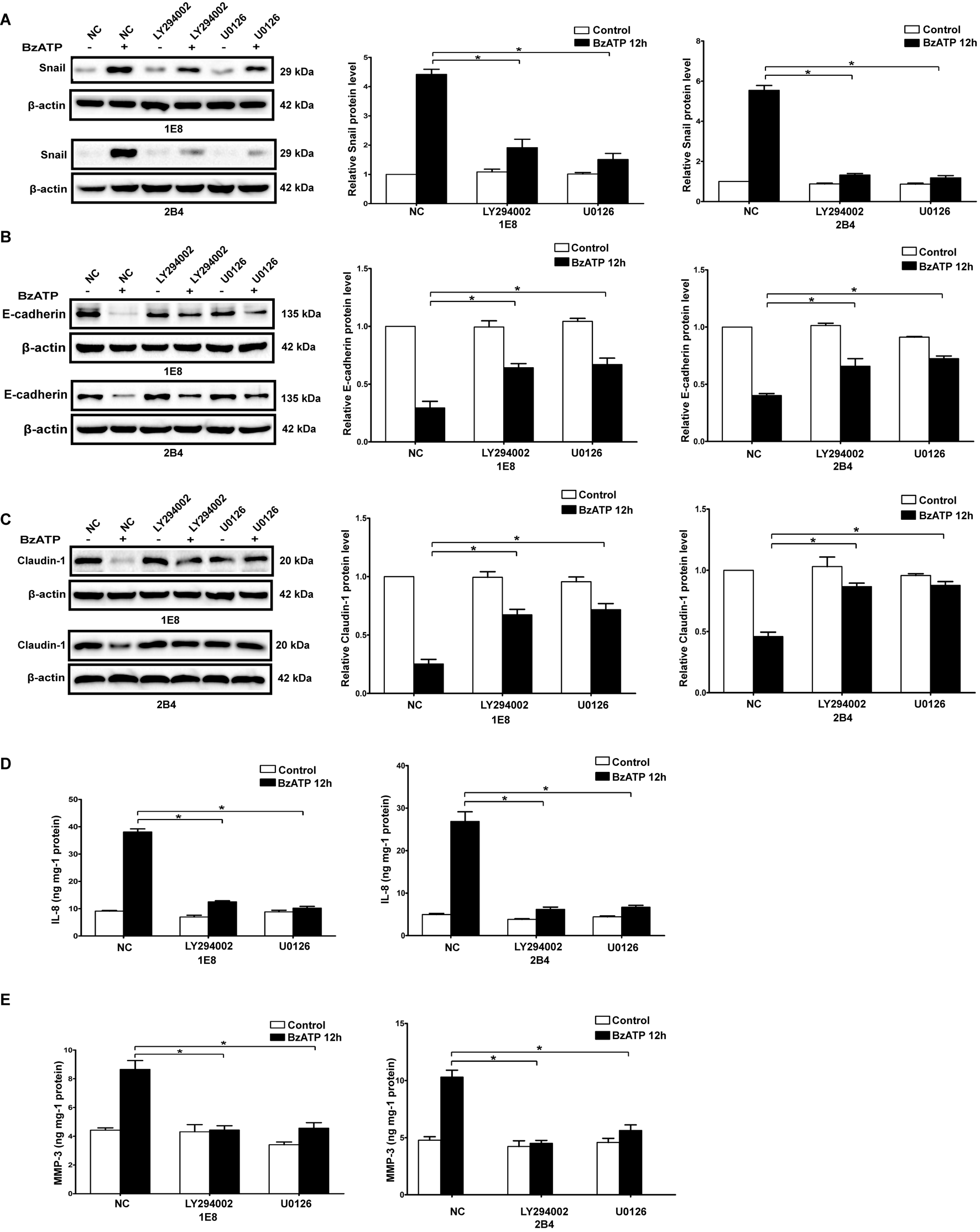

Supplement: S7 Figure — Effects of PI3K/AKT and ERK1/2 signaling pathways on BzATP-induced expression changes of EMT/invasion-related genes. IE8 and 2B4 cells were treated with LY294002 (lanes denoted as LY294002) or U0126 (lanes denoted as U0126) or without treatment (served as a negative control, lanes denoted as NC). Expressions of Snail (A), E-cadherin (B) and Claudin-1 (C) were detected by western blots. Expression of IL-8 (D) and MMP-3 (E) were detected using ELISA. Expressions of these proteins were normalized to their respective expression in control cells (without BzATP). Data were presented as mean ± s.d. (vertical bars). At least three independent experiments were performed. *P<0.05. (TIF) [file pone.0114371.s007.tif]

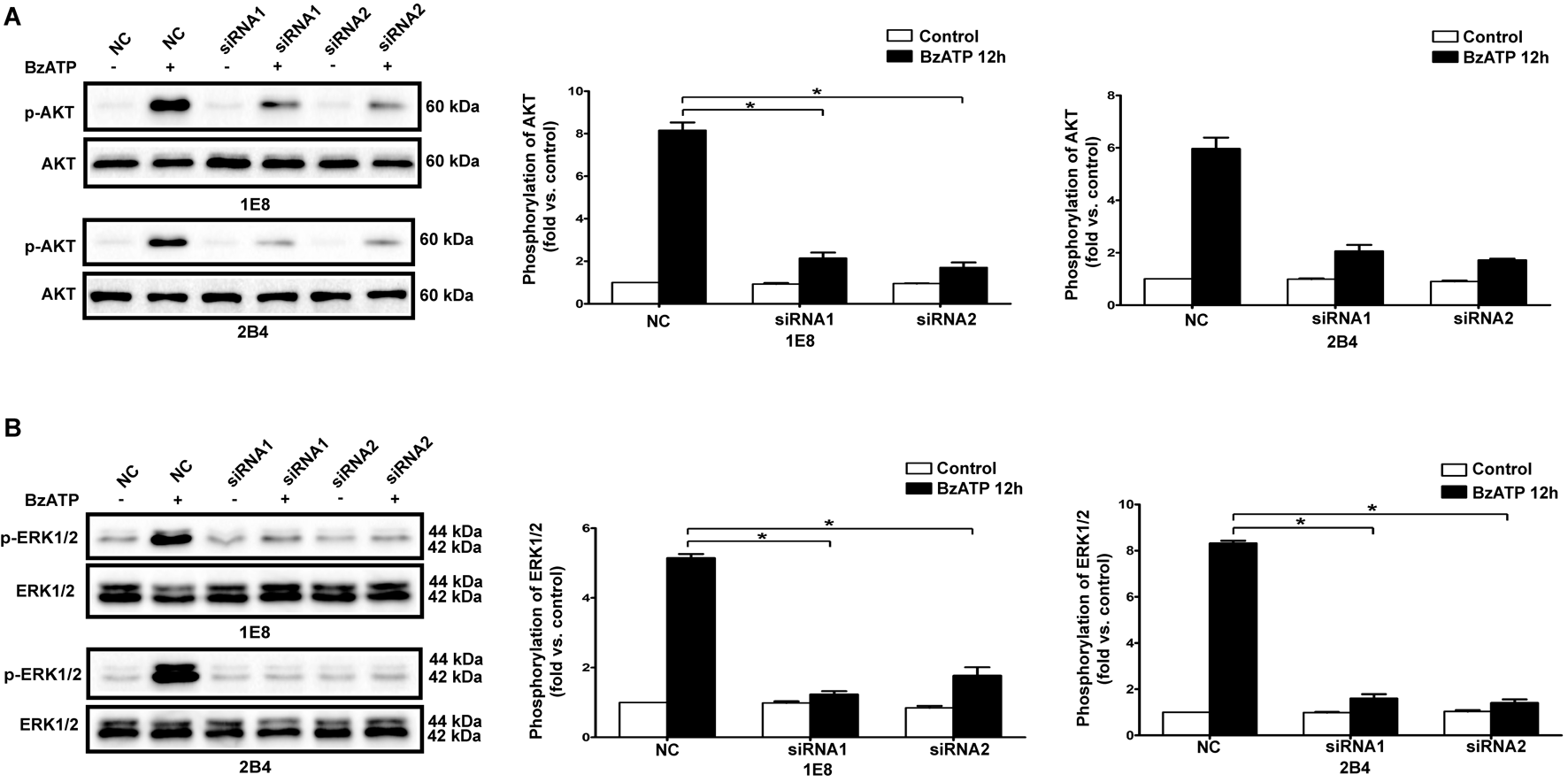

Supplement: S8 Figure — Knockdown of P2X7 attenuated BzATP-mediated activation of PI3K/AKT and ERK1/2 signaling pathways. P2X7 silenced cells (siRNA1 and siRNA2) and control siRNA cells (NC) were treated with or without 100 µM BzATP for 15 min. Western blot experiments were performed to analyze phosphorylation level of AKT (A) and ERK1/2 (B). Expression of p-AKT and p-ERK1/2 were normalized to their respective expression in control cells (without BzATP). Data were presented as mean ± s.d. (vertical bars). At least three independent experiments were performed. *P<0.05. (TIF) [file pone.0114371.s008.tif]
