# Supplementary material for: P2X7 Mediates ATP-Driven Invasiveness in Prostate Cancer Cells
Source: PLoS One. 2014 Dec 8;9(12):e114371. doi: 10.1371/journal.pone.0114371 (PMC4259308; doi:10.1371/journal.pone.0114371)
Supplement: S1 Table — Sequence of the primers used in the real-time qPCR experiments. (DOC) [file pone.0114371.s009.doc]

**Supplementary Table 1 Sequence of the primers used in the real-time qPCR experiments.**

| Gene | Primer sequence | PCR product  size(bp) |
| --- | --- | --- |
| Snail | Forward: 5'-AATCGGAAGCCTAACTACAGCG-3' | 147 |
|  | Reverse: 5'-GTCCCAGATGAGCATTGGCA-3' |  |
| E-cadherin | Forward: 5'-CTGGGCTGGACCGAGAGA-3' | 60 |
|  | Reverse: 5'-GAAGGTCAGCAGCTTGAACCA-3' |  |
| Claudin-1 | Forward: 5'-TGAAGTGCTTGGAAGACGATG-3' | 95 |
|  | Reverse: 5'-GGCAACTAAAATAGCCAGACCT-3' |  |
| IL-8 | Forward: 5'-ACTGAGAGTGATTGAGAGTGGAC-3' | 112 |
|  | Reverse: 5'-AACCCTCTGCACCCAGTTTTC-3' |  |
| MMP-3 | Forward: 5'-ATGGACAAAGGATACAACAGGGA-3' | 127 |
|  | Reverse: 5'-TGTGAGTGAGTGATAGAGTGGG-3' |  |
| P2X7 | Forward: 5'- TATGAGACGAACAAAGTCACTCG -3' | 95 |
|  | Reverse: 5'- GCAAAGCAAACGTAGGAAAAGAT -3' |  |
| β-actin | Forward: 5'-GGATGCAGAAGGAGATCACTG-3' | 90 |
|  | Reverse: 5'-CGATCCACACGGAGTACTTG-3' |  |
